# Inflammatory endothelium-targeted and cathepsin responsive nanoparticles are effective against atherosclerosis

**DOI:** 10.7150/thno.70896

**Published:** 2022-05-16

**Authors:** Fei Fang, Yinghao Ni, Hongchi Yu, Hongmei Yin, Fan Yang, Chunli Li, Denglian Sun, Tong Pei, Jia Ma, Li Deng, Huaiyi Zhang, Guixue Wang, Song Li, Yang Shen, Xiaoheng Liu

**Affiliations:** 1 Institute of Biomedical Engineering, West China School of Basic Medical Sciences & Forensic Medicine, Sichuan University, Chengdu 610041, China.; 2 Department of Bioengineering and Department of Medicine, University of California, Los Angeles, Los Angeles 90001, USA.; 3 West China School of Pharmacy, Sichuan University, Chengdu 610041, China.; 4 Key Laboratory for Biorheological Science and Technology of Ministry of Education, State and Local Joint Engineering Laboratory for Vascular Implants, College of Bioengineering, Chongqing University, Chongqing 400030, China.

**Keywords:** atherosclerosis, cathepsin k, nanoparticles, rapamycin, drug delivery

## Abstract

**Rationale:** Atherosclerosis is characterized by lipid accumulation, plaque formation, and artery stenosis. The pharmacological treatment is a promising therapy for atherosclerosis, but this approach faces major challenges such as targeted drug delivery, controlled release, and non-specific clearance.

**Methods:** Based on the finding that the cathepsin k (CTSK) enzyme is enriched in atherosclerotic lesions, we constructed an integrin α_v_β_3_ targeted and CTSK-responsive nanoparticle to control the release of rapamycin (RAP) locally. The targeted and responsive nanoparticles (T/R NPs) were engineered by the self-assembly of a targeting polymer PLGA-PEG-c(RGDfC) and a CTSK-sensitive polymer PLGA-Pep-PEG. PLGA-Pep-PEG was also modified with a pair of FRET probe to monitor the hydrolysis events.

**Results:** Our results indicated that RAP@T/R NPs accelerated the release of RAP in response to CTSK stimulation* in vitro*, which significantly inhibited the phagocytosis of OxLDL and the release of cytokines by inflammatory macrophages. Additionally, T/R NPs had prolonged blood retention time and increased accumulation in the early and late stage of atherosclerosis lesions. RAP@T/R NPs significantly blocked the development of atherosclerosis and suppressed the systemic and local inflammation in ApoE^-/-^ mice.

**Conclusions:** RAP@T/R NPs hold a great promise as a drug delivery system for safer and more efficient therapy of atherosclerosis.

## Introduction

Atherosclerosis is a chronic inflammatory disease characterized by the accumulation of lipids, immune cells and inflammatory cytokines in the vascular intima 1. Traditional oral medications (including statins, antiplatelet, and vasodilators) have limitations for atherosclerosis therapy due to their non-specific distribution and poor water solubility 2. In addition, long-term treatment may cause serious side effects, such as liver damage, muscle pain, and diabetes mellitus 3.

Nanocarriers approach has been demonstrated its promise for atherosclerosis therapy. Nanoparticles could effectively target the lesion sites by binding with different receptors, such as intercellular cell adhesion molecule 1 (ICAM-1), vascular cell adhesion protein 1 (VCAM-1), integrin α_v_β_3_, and p-selectin 4. However, considerable challenges remain in targeting efficiency and controllable drug release at the lesion sites 5. To address this unmet need, we developed a novel nanocarrier that integrates targeting and responsive drug delivery for atherosclerotic lesions.

Compared with healthy arteries, atherosclerosis lesions exhibit high reactive oxygen species (ROS) 6-8 and lower pH values 7, 9. In addition, atherosclerosis is enriched with matrix metalloproteinases (MMPs) 10 and cysteine proteases 11, which are involved in extracellular matrix (ECM) remodeling. Recent evidence from human tissues and preclinical animal models indicated that cysteine protease cathepsins played an essential role in developing vascular diseases 12. Cathepsin K (CTSK) is expressed in vascular endothelial cells (VECs), vascular smooth muscle cells (VSMCs), and macrophages 13. CTSK is mainly involved in the elastic intima's hydrolysis at the early stage of atherosclerosis, contributing to VSMCs proliferation 14. At the late stage of atherosclerosis, CTSK could hydrolyze the ECM components in the necrotic nucleus, resulting in vulnerable plaques 15. The expression of CTSK is regulated by cytokines 16, 17, cholesterol, and oxidized LDL (OxLDL) 18. It was also found that oscillating shear stress could induce CTSK expression and the level of CTSK in atherosclerotic plaque was significantly higher than that in healthy tissues 19. Consistently, atherosclerotic plaques predominantly form in the regions with low or oscillating shear stress. In addition, local disturbed flow forms near the shoulder of the atherosclerotic plaque, which may further promote the expression of CTSK and aggravate atherosclerosis. Furthermore, pro-CTSK (the precursor of CTSK) only exposes the protease's active domain under acidic conditions, suggesting that CTSK has optimal activity under acidic pH 20. It has been reported that the atherosclerotic plaque microenvironment is weakly acidic, with a pH range of pH 6.0-6.8 21. Another study indicated that the extracellular microenvironment's acidification (a pH value of 5.5) occurs in advanced atherosclerotic plaques 22. Therefore, CTSK could be an ideal candidate to trigger drug release from nanocarriers in atherosclerosis lesions.

In this study, we designed CTSK-sensitive and integrin-targeted nanoparticles to deliver rapamycin (RAP) for the treatment of atherosclerosis. As the specific hydrolysis substrate of CTSK, the peptide sequence HPGGPQ (Dabcyl-Lys-HPGGPQ-Glu (EDANS)-acp-Cys-NH_2_) of type I collagen was designed and synthesized 23, 24. We introduced this peptide sequence between the hydrophobic polymer poly (lactic-co-glycolic acid) (PLGA5000, 50:50) and the hydrophilic polymer poly (ethylene glycol) (PEG2000) to form a block copolymer PLGA-Pep-PEG. Moreover, targeting polymers (PLGA-PEG-c(RGDfC)) was made by covalently linking PLGA-PEG-MAL to an α_v_β_3_ targeting peptide c(RGDfC). Finally, the targeted and responsive nanoparticles (T/R NPs) were generated through the self-assembly of PLGA-Pep-PEG and PLGA-PEG-c(RGDfC) to encapsulate RAP. The RAP@T/R NPs could target atherosclerotic lesions by binding to the overexpressed α_v_β_3_ in inflammatory VECs, and disintegrate and release RAP in response to CTSK. Our study demonstrated that the RAP@T/R NPs effectively accumulated in the early and late stage of atherosclerosis lesions, reduced inflammation, and inhibited atherosclerosis development.

## Materials and Methods

### Materials

Poly (lactic-co-glycolic acid, 50:50)-maleimide (PLGA_5000_-MAL, Catalog # R-PL1182-5K), Poly (lactic-co-glycolic acid, 50:50)-poly (ethylene glycol)-maleimide (PLGA_5000_-PEG_2000_-MAL, Catalog # R-PL1003-7KD) and Poly (lactic-co-glycolic acid, 50:50)-poly (ethylene glycol) (PLGA_5000_-PEG_2000_, Catalog # R-PL1001-7KD) were obtained from the Xi'an ruixi Biological Technology Co., Ltd (Xian, China). Poly (ethylene glycol)-N-hydroxysuccinimide (PEG_2000_-NHS, Catalog # E03181) was obtained from Shanghai Ponsure Biotech, Inc. The c(RGDfC) peptide and CTSK sensitive peptide Dabcyl-Lys-HPGGPQ-Glu (EDANS)-acp-Cys-NH_2_ were custom-synthesized by BankPeptide Inc. (Hefei, China). Human recombinant CTSK protein (Catalog # 219461) and lipopolysaccharide (LPS) (Catalog # L2880) were purchased from Merck (China). Rapamycin (Catalog # C11382414) and Brij-58 (Catalog, # B124616) were acquired from Macklin Inc. (Shanghai, China). The DIR dye (Catalog # D4006) was purchased from Suzhou Yuheng Biotechnology Co., Ltd. (Suzhou, China). Cell Counting Kit-8 (CCK-8 kit, Catalog # CK04) from Dojindo Laboratories. (Shanghai, China). Dil-OxLDL (Catalog # YB-0010) was purchased from Guangzhou Yiyuan Biotech Co., Ltd. (Guangzhou, China). LysoTracker (Catalog # C1047S) was obtained from the Beyotime Institute of Biotechnology. (Shanghai, China). The FITC labeled phalloidin (Catalog # CA1620), dithiothreitol (Catalog # B8220), and Masson's Trichrome Stain Kit (Catalog # G1340) were purchased from Solarbio Molecular Technologies (Beijing, China). ELISA kits to assay the levels of mouse tumor necrosis factor-α (TNF-α) (Catalog # JYM0218Mo), interleukin-1β (IL-1β) (Catalog # JYM0531Mo), and monocyte chemotactic protein-1 (MCP-1) (Catalog # JYM0099Mo) were obtained from Jymbio, Colorful Gene Biological Technology Co. Ltd. (Wuhan, China). The antibodies CTSK (Catalog # DF6641) was obtained from Affinity Biosciences Ltd. (USA), antibodies integrin α_v_ (Catalog # SC376156), integrin β_3_ (Catalog # SC365679), was obtained from Santa Cruz Biotechnology, Inc. (USA), antibody CD31 (Catalog # ab281583), MMP-9 (Catalog # ab228402), CD68 (Catalog # ab125212), α-SMA (Catalog # ab32575), were purchased from Abcam (Shanghai, China).

### Animals

Male C57BL/6 mice and male apolipoprotein E knockout (ApoE^-/-^) mice (six-week-old) were obtained from the GemPharmatech Co., Ltd. Animals were housed in standard mice cages with ad libitum access to water and food. Before experiments, all mice were acclimatized for at least three days. All animal work was performed under the guidelines of the China Council on Animal Care and Sichuan University protocol for animal use. All ethical guidelines for experimental animals were followed.

### Histological assays for CTSK and integrin α_v_ in ApoE^-/-^ mice

ApoE^-/-^ mice were fed a high-fat diet for 12 weeks, the healthy C57BL/6 mice as the control groups. All mice were euthanized and then perfused with 10 mL of heparin sodium to remove blood. After careful dissection of the tissue surrounding the abdominal aorta, it was fixed with 4% paraformaldehyde for 4 hours. Next, the samples were prepared into 10-μm thick paraffin sections. For immunofluorescence experiments, samples were deparaffinized with xylene, treated with graded alcohol, and then blocked with 5% goat serum for 2 hours. Next, different primary antibodies (including CTSK (1:300) or integrin α_v_ (1:300)) were incubated overnight. After incubation with FITC-conjugated secondary antibodies, nuclei were labeled with DAPI dye. Finally, the samples were observed and photographed by confocal laser scanning microscopy (CLSM) (Zeiss, LSM710).

For immunohistochemistry (IHC) analysis, samples were deparaffinized with xylene and treated with graded alcohol. Subsequently, the sections were incubated with 3% H_2_O_2_ for 15 minutes to eliminate endogenous peroxidase and boiled in citrate buffer for 10 minutes. Next, the sections were blocked with 5% goat serum for 2 hours and then incubated with different primary antibodies (including CTSK (1:300) or integrin α_v_ (1:300)) overnight. At room temperature, samples were incubated with biotinylated secondary antibodies for 2 hours, followed by horseradish enzyme-labeled streptavidin for 1 hour. After incubation with DAB chromogenic solution for 5 minutes, the nuclei were stained with hematoxylin. Finally, the samples were observed by a microscope slide scanner (Olympus Optical Co Ltd, Japan). The positively stained areas of each sample were measured using the ImagePro Plus 6.0 software (National Institutes of Health, Bethesda, USA) 25.

### Partial carotid ligation models

The partial carotid ligation model of normolipidemic mice was performed as described previously 26. Briefly, the external carotid artery (ECA), occipital artery (OA), and internal carotid artery (ICA) of the left carotid artery (LCA) were ligated, leaving the superior thyroid artery (STA) intact. Unligated right carotid artery (RCA) was used as a control. These mice were fed with a standard lab chow diet for 21 days, which could create disturbed blood flow at the LCA and aortic arch (AA) sites, and induce the endothelial dysfunction phenotype of initial atherosclerosis *in vivo*.

### *En face* immunostaining

Mature C57BL/6 mice (weighing about 25 ± 2g) were subjected to partial carotid ligation for 21 days. After sacrificing the mice, aortic tissues, including LCA, RCA, AA, and TA, were collected. Then, *En face* immunostaining was performed on the luminal side of the aortas and common carotid arteries as described previously 27. Briefly, dissected tissues were fixed in 4% paraformaldehyde for 4 hours and washed with 0.3% PBST (0.3% Triton X-100 in PBS) 3 times (15-minute each time) at room temperature. After washing, samples were blocked with 5% goat serum at 4 °C overnight. The LCA, RCA, AA, and TA were incubated with antibodies for immunofluorescence analysis, including CTSK (1:300) and CD31 (1:500) at 4 °C overnight. Subsequently, these samples were incubated with corresponding fluorescently labeled secondary antibodies for 2 hours at room temperature. The nuclei were stained with DAPI dyes and observed under a CLSM.

### Immunofluorescence staining for CTSK and integrins α_v_β_3_ in ligation mice

Mature C57BL/6 mice (weighing about 25 ± 2g) were subjected to partial carotid ligation for 21 days. After sacrificing the mice, the 10-μm thick frozen sections of LCA, RCA, TA, and AA were prepared. For immunofluorescence staining, tissue sections of RCA, LCA, TA and AA were fixed in 4% paraformaldehyde for 4 hours. After blocking in 5% goat serum in PBS for 2 hours, the sections were immunostained with the antibodies as indicated, including CD31 (1:500), CTSK (1:300), integrin α_v_ (1:300), and integrin β_3_ (1:300) at 4 °C overnight. Subsequently, these samples were incubated with corresponding fluorescently labeled secondary antibodies for 2 hours at room temperature. The nuclei were stained with DAPI dyes and observed under a CLSM.

### Synthesis of CTSK responsive block polymer PLGA-Pep-PEG

The CTSK sensitive peptide Dabcyl-Lys-HPGGPQ-Glu(EDANS)-acp-Cys-NH_2_ was custom-synthesized by BankPeptide Inc. (Hefei, China). PLGA_5000_-MAL and CTSK responsive peptides Dabcyl-Lys-HPGGPQ-Glu(EDANS)-acp-Cys-NH_2_ (molar ratio 1:1.2) were dissolved in DMF and adjusted the pH to 7.2 with triethylamine. After stirring gently overnight at 4 °C, the free peptide was removed by dialysis bag (MWCO = 3500). Then, freeze-dry the liquid in the dialysis bag to obtain the PLGA-Pep conjugates. Similarly, the obtained PLGA-Pep and PEG_2000_-NHS (molar ratio of 1.2:1) were dissolved in DMF and adjusted pH value to 8.0 with triethylamine. After reacting overnight at 4 °C, remove the free PEG_2000_-NHS by dialysis bag (MWCO=7500). PLGA-Pep-PEG is obtained after freeze-drying.

### Synthesis of targeted block polymer PLGA-PEG-c(RGDfC)

The PLGA_5000_-PEG_2000_-MAL and c(RGDfC) peptide (molar ratio 1:1.2) were dissolved in DMF, and the pH was adjusted to 7.2 with triethylamine, stirred gently at 4 °C overnight. Subsequently, the free c(RGDfC) peptides were removed using a dialysis bag (MWCO = 7500). PLGA-PEG-c(RGDfC) was obtained after freeze-drying.

### Characterizations of PLGA-Pep-PEG and PLGA-PEG-c(RGDfC)

The chemical compositions of PLGA-Pep-PEG and PLGA-PEG-c(RGDfC) were analyzed by Fourier transform infrared spectroscopy (FT-IR, Nicolet 380, Thermo Electrics) and ^1^H nuclear magnetic resonance spectra (NMR, Avance III 400, Bruker). In addition, the number average molecular weight (Mn) and weight average molecular weight (Mw) distribution of the polymers were measured by gel permeation chromatography (GPC, Shimadzu LC20) with a differential refractometer as the detector (Shimadzu DIR-20). DMF was used as the elution solvent with a 1.0 mL/minute flow rate at 35 °C, and PEG was used as the standard.

### Preparation of nanoparticles

RAP@T/R NPs were made by using the classical emulsion-solvent evaporation method. Briefly, PLGA-Pep-PEG and PLGA-PEG-c(RGDfC) were dissolved in chloroform at a mass ratio of 1:1, and various amounts of RAP (same, 50% or 25% of the polymer mass) were added. After thoroughly stirring, we used a micro-injector to drip the mixture solution into an aqueous solution of 10 times the volume of the mixture at a rate of 2 mL/hour. The dispersed micelles were sonicated for 10 minutes and filtered using a 0.45 μm microporous membrane. After that, the powder of T/R NPs was obtained by freeze-drying the filtered solution. Similarly, the RAP@NPs (RAP loaded PLGA-PEG nanoparticles), DIR@NPs (DIR loaded PLGA-PEG nanoparticles), DIR@R NPs (DIR loaded PLGA-Pep-PEG nanoparticles), and DIR@T/R NPs were prepared by the above method.

### Characterization of nanoparticles

The morphology of T/R nanoparticles was observed by a transmission electron microscope (TEM). The zeta potentials and hydrated particles' diameter of T/R NPs were analyzed using a dynamic light scattering (DLS) instrument. Differential scanning calorimetry (DSC) was used to detect the weight loss curves of RAP, unloaded T/R NPs, and RAP@T/R NPs, respectively. The temperature range of 30-200 °C, the heating rate of 10 °C/minute, and the nitrogen flow rate of 1 mL/minute. To assess nanoparticles stability *in vitro*, T/R NPs and RAP@T/R NPs were incubated with 10% fetal bovine serum in PBS, and the hydrodynamic sizes were measured at 0, 24, 48, and 72 hours by DLS.

### *In vitro* study of enzymolysis

CTSK activation was performed in 0.1 M acetate buffer solution (pH 5.5), including 2.5 mM EDTA and 2.5 mM dithiothreitol. Then, 50 μL, 1 mg/mL of responsive polymer or nanoparticles were incubated with 10 nM CTSK enzyme in a buffer solution. The buffer solution and CTSK antibody (1:200) were negative and positive control groups. The generated EDANS fluorescence from CTSK sensitive peptide was measured with a fluorescence microplate reader using excitation at 336 nm and emission at 490 nm for 56 hours.

### Drug loading and drug release *in vitro*

The RAP amount in the T/R NPs was measured by high-performance liquid chromatography (HPLC) (Shimadzu LC-2030C Plus) with a reversed-phase column (4.6 × 250 mm, 5 μm). The mobile phase comprised acetonitrile and water with a volume ratio of 7:3, and the flow rate was set as 1.2 mL/minute. The column temperature was 55 °C, and the detection wavelength was 278 nm. The drug loading content (DLC) and drug encapsulation efficiency (DEE) of RAP@T/R NPs were calculated using Eqs. (1) and (2), respectively.

DLC (%) = 
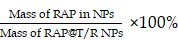

(1)

DEE (%) = 
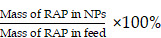

(2)

The release profile of RAP in T/R NPs was studied with the addition of CTSK (0, 10 nM, respectively). T/R NPs (1 mg) dispersed in 1 mL PBS (pH7.4, contains 0.1% Brij-58 as a surfactant to improve the solubility and stability of RAP) or acetate buffer solution (pH 5.5, contains 0.1% Brij-58) were loaded in a dialysis bag (MWCO = 7500) with 20 mL solution, and shake at 37 °C. At various time points, 1 mL of buffer solution was taken out for sampling, and an equivalent amount of fresh buffer solution was supplemented. HPLC was used to quantify the cumulative release of RAP in T/R NPs.

### Cell cytotoxicity evaluation *in vitro*

Human umbilical vein vessel endothelial cells (HUVECs), human arterial smooth muscle cells (HASMCs), and RAW264.7 cells (purchased from ATCC, USA) were cultured in the 96-well plates at a density of 5
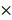
10^3^ cells per well until 90% confluence. T/R NPs of different concentrations (0, 50, 100, 200, 300, 400, 500 μg/mL) were added and incubated for 24 hours. After changing the fresh medium, 10 μL of CCK-8 reagent was added to each well, incubated for another 4 hours, and then measured the absorbance at 450 nm.

### *In vitro* blood compatibility tests

For hemolysis assay, the red blood cells (RBCs) were obtained from the whole blood of C57BL/6 mice. 10% RBCs (v/v, in PBS) were incubated with different concentration (0, 50, 100, 200, 300, 400, 500 μg/mL) of T/R NPs at 37 °C for 3 hours. Centrifuge at 10000g for 1 minute, the supernatant was taken out for analysis by a microplate reader at 541 nm. The hemolytic percentage was calculated by Eqs. 3.

Hemolytic percentage (%) = 
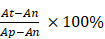

(3)

At, An, and Ap are the absorbance values of samples, negative control, and positive control. 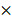
1 PBS as negative control and deionized water as a positive control, respectively.

### Cell uptake

HUVECs were cultured into a 24-well plate with approximately 5
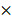
10^4^ cells per well. After changing the fresh medium, 50 μg/mL of T/R NPs were added and incubated for different times (0.5, 1, 2, 4, 6, and 8 h). After removing the T/R NPs, cells were fixed with 4% paraformaldehyde and blocked with 5% goat serum for 2 hours. Subsequently, the cells were incubated with CD31 antibody (1:300) overnight and incubated with FITC-conjugated second antibody for 2 hours. The nuclei were stained with DAP and observed with a CLSM.

### Flow cytometric analysis of cellular uptake of T/R NPs

HUVECs (1
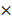
10^6^ cells/well) were cultured in 6-well plates for 24 hours. Then, incubate HUVECs with 100 μg/mL of DIR@T/R NPs for a predefined time (0.5, 1, 2, 4, 6, and 8 hours). HUVECs without DIR@T/R NPs treatment as the control group. The cells were washed with PBS three times and harvested for flow cytometric analysis (BD Accuri C6). Results were analyzed using FlowJo software (version 10.0.7r2).

### Evaluation of T/R NPs targeting ability* in vitro*

HUVECs (5×10^4^ cells/well) were seeded in the 24-well plates for 12 hours. Then, 500 ng/mL LPS was added to activate the cells for 24 hours. In the experimental group, 5 μM of cilengitide and 1 μg/mL of c(RGDfC) peptide were added to pre-incubate for 2 hours, and then 50 μg/mL of DIR@T/R NPs, DIR@NPs, and DIR@R NPs were added to incubate for another 2 hours, respectively. The cells were washed with PBS three times and fixed with 4% paraformaldehyde. The cells were incubated with CD31 antibody (1:300) overnight, and FITC-conjugated the second antibody was incubated for 2 hours. The nuclei of the cells were stained with DAPI, and the samples were observed using CLSM.

### Evaluation of translocated of T/R NPs by transwell system

The transwell model was established by incubating monolayer HUVECs in the upper chamber and the monolayer RAW264.7 cells in the bottom chamber. Then, 500 ng/mL LPS was added to the top chamber to stimulate HUVECs for 24 hours. 100 μg/mL of DIR@T/R NPs, DIR@NPs, or DIR@R NPs were added to the upper chamber incubated for 4 hours. RAW264.7 cells in the bottom chamber were washed by PBS three times and fixed with 4% paraformaldehyde for 30 minutes. The F-actin and nuclei of the cells were stained with FITC-phalloidin and DAPI, followed by microscopy using CLSM.

### Inhibition of foam cell formation of RAP@T/R NPs

RAW264.7 cells were cultured in 24-well plates at 1
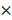
10^3^ cells per well. After stimulation with 500 ng/mL of LPS for 24 hours, the cell was incubated with 50 μg/mL of RAP@T/R NPs or 50 μg/mL of RAP@NPs or 5 μg/mL of free RAP for 2 hours. Cells were subsequently stimulated with 500 ng/mL of LPS for 24 hours, and an equal amount of PBS was added to the control groups. Then, 30 μg/mL of Dil-OxLDL was added and incubated for 4 hours. After washing three times with PBS, RAW264.7 cells were stained with phalloidin and DAPI, respectively. Then, the samples were observed by CLSM. In addition, quantification analysis was performed by flow cytometric.

### Evaluation of *in vitro* anti-inflammatory effects

RAW264.7 cells were seeded in 24-well plates at 1
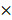
10^4^ cells per well. These cells were incubated with 50 μg/mL of RAP@T/R NPs, 50 μg/mL of RAP@NPs, or 5 μg/mL of RAP or an equivalent amount of PBS (as the control) for 2 hours. Cells were subsequently stimulated with 500 ng/mL LPS for 24 hours. Then, typical inflammatory factors are detected by ELISA kits, including TNF-α, IL-1β, and MCP-1.

### Inhibition of proliferation of macrophages *in vitro*

RAW264.7 cells were cultured in 96-well plates with a density of 1
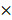
10^3^ cells for each well. After 12 hours, 5 μg/mL of RAP, 50 μg/mL of RAP@NPs or 50 μg/mL of RAP@T/R NPs were incubated with cells for 24 hours. Subsequently, cell proliferation was detected by the CCK-8 assay.

### Acute toxicity evaluation of nanoparticles

Healthy C57BL/6 mice (weighing about 25 ± 2g) were injected with saline and 5 mg/kg of T/R NPs or NPs. After 24 hours, the mice were sacrificed, and whole blood and serum were collected for a completed blood panel and blood biochemical characteristics test. The main characteristics include RBCs, white blood cells (WBCs), platelets (PLT), lymphocytes, neutrophil granulocyte (GRAN), hemoglobin (HGB), aminotransferase (ALT), alkaline phosphatase (ALP), albumin (ALB), γ-glutamyl transferase (γ-GT), creatinine (CREA), UREA, uric acid (UA), and total bile acid (TBA). In addition, the major organs, including the heart, liver, kidney, lung, and spleen, were harvested for hematoxylin-eosin (H&E) staining analysis.

### *In vivo* pharmacokinetic study

Mature C57BL/6 mice (weighing about 25 ± 2g) are used as experimental models. DIR@T/R NPs were injected into mice by tail vein at a 100 mg/kg dose. 100 mg/kg of DIR@NPs and the same dose of saline were injected as positive and negative groups, respectively. At predefined time points (0.5, 1, 3, 6, 12, 24, 36, 48 hours), 20 μL of fresh blood taken from the fundus venous plexus of mice was mixed with 10% heparin sodium. Then, the mixture was transferred to a 96-well fluorescence microtiter plate. Using the *in vivo* image system (IVIS) (Perkin Elmer, USA), the mean fluorescence intensities (MFI) of blood samples were determined.

### Evaluation of the targeting effect of T/R NPs *in vivo*

Six-week-old ApoE^-/-^ mice were fed high-fat food for 12 weeks. The 100 mg/kg of DIR@T/R NPs were tail vein injection into mice, 100 mg/kg of DIR@NPs and saline treatment as a control group. After 6 hours, mice were euthanized and then perfused with 10 mL of heparin sodium to remove unbound DIR-loaded nanoparticles. The aortas and major organs, including the heart, liver, kidney, lung, and spleen, were fixed with 4% paraformaldehyde and observed using IVIS. The AA was also sliced into 10-μm thick slices after embedding with OCT. The samples were blocked 10% normal goat serum for 60 minutes and incubated with CD31 antibody (1:300), or CD68 antibody (1:300) in 10% normal goat serum in the dark at 4 °C overnight. Subsequently, the samples were incubated with 1:500 diluted FITC-labeled second antibody in 10% normal goat serum at room temperature for 2 hours and counterstained with DAPI. Finally, CLSM was employed to observe these samples.

Adult C57BL/6 mice (weighing about 25 ± 2g) were subjected to partial carotid ligation for 21 days. 100 mg/kg of DIR@T/R NPs were tail vein injections into mice, 100 mg/kg of DIR@NPs treatment as a control group. After 6 hours, mice were euthanized and then perfused with 10 mL of heparin sodium to remove unbound DIR-loaded nanoparticles. Then, isolate the aortas for imaging and quantitative analysis using an IVIS.

### Treatment of atherosclerosis in ApoE^-/-^ mice

Six-week-old ApoE^-/-^ mice were fed high-fat food for 8 weeks and randomly assigned into four groups (*n* = 15) subjected to various treatments for additional 4 weeks. The control group mice were subjected to tail vein injection with saline. The other groups were given *i.v.* with free RAP, RAP@NPs, and RAP@T/R NPs at the dose of 0.5 mg/kg of RAP twice a week. During treatment, the bodyweight of mice was monitored regularly.

### Quantification of atherosclerotic plaques

After administration for 4 weeks, all mice were euthanized, and the aorta and heart were isolated. Aorta and heart were resected and perfused with 10% neutral buffered formalin for 4 hours. Carefully removing the periadventitial tissue and dissecting the aorta longitudinally, the atherosclerotic plaques of the aorta were detected by enface Oil Red O (ORO) staining. In addition, 4-5 serial 10-μm thick cross-sections of the aortic root, AA (between the brachiocephalic artery and LCA), and abdominal aorta (about 5-mm below the renal artery) were prepared and stained with ORO. Quantitative analysis of atherosclerotic plaque areas was determined by software ImagePro Plus 6.0 software.

### Histology and IHC staining for aorta tissues

After being fixed in 4% paraformaldehyde for 4 hours, the serial 10-μm thick aorta tissue paraffin sections, including aortic root, AA (between the brachiocephalic artery and LCA), TA (about 10 mm below the LSA), abdominal aorta (about 5 mm below the renal artery), and brachiocephalic artery (about 2 mm below the RSA), were prepared with H&E, Masson's trichrome, and IHC staining (*n* = 30). For IHC analysis, the aorta tissue sections were incubated with different antibodies, including MMP-9 (1:500), CTSK (1:300), CD68 (1:300), and α-SMA (1:500) at 4 °C overnight, respectively. The lesions, collagen-positive areas, and IHC positively stained areas of each aorta area were measured using the ImagePro Plus 6.0 software.

### Quantification of inflammatory cytokines in the aortic tissue and serum

After different treatments, the mice were sacrificed, and the aorta and serum were collected. After the aortic tissue was minced, 100 μL of PBS was added and fully homogenized. Then, the supernatant was obtained by centrifugation at 10000 g for 10 minutes. Inflammatory factors (including TNF-α, IL-1β, and MCP-1) in the aorta and serum were quantitated by ELISA kits.

### Pharmacokinetic profiles of RAP in the aorta

After 72 hours of drug administration, mice were euthanized, and the aorta was isolated. The aorta was minced and homogenized in 0.2 mL of saline. Then, 0.2 mL of sodium carbonate was added and vortexed for 1 minute. After vortexing, 10 mL of tert-butyl methyl ether was added and vortexed for 15 minutes to extract rapamycin. The supernatant was obtained by centrifugation at 5000 rpm for 5 minutes. The residual samples were dissolved in 100 μL methanol solution and analyzed by HPLC (Shimadzu LC-2030C Plus) with a reversed-phase column (4.6 × 250 mm, 5 μm). The mobile phase comprised acetonitrile and water with a volume ratio of 7:3, and the flow rate was set as 1.2 mL/minute. The column temperature was 55 °C, and the detection wavelength was 278 nm.

### Safety evaluation

The mice were sacrificed 12 hours after the last injection, and the whole blood and serum were collected for a completed blood panel and blood biochemical characteristics test. The main characteristics include RBCs, WBCs, PLT, GRAN, HGB, ALT, ALP, ALB, γ-GT, CREA, UREA, UA, and TBA. The major organs (heart, liver, spleen, lung, and kidney) were sectioned and analyzed by H&E staining.

### Statistical analysis

All data are expressed as the mean ± standard deviation (SD) in this study. IBM SPSS Statistics 26 software (IBM, USA) was used for the statistical analysis. One-way ANOVA and Student's t-test were utilized for statistical analysis.

### Data availability

The authors declare that all data supporting the findings of this study are included within the article and its Supplementary Information, and are also available from the authors upon request.

## Results

### The expression of CTSK, integrin α_v_ in the early and late stages of atherosclerosis

**Figure 1:**
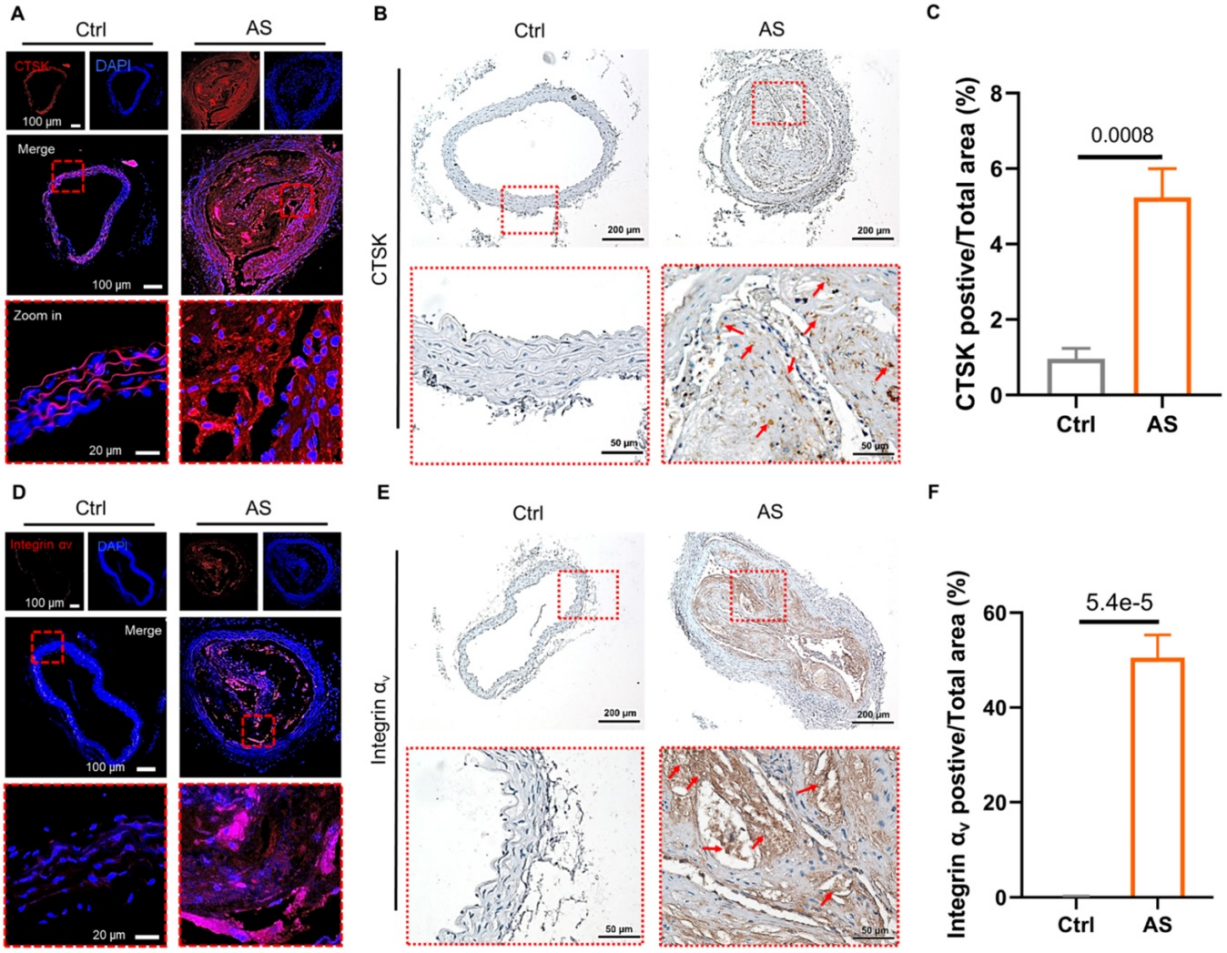
** Histological assays for CTSK and integrin α_v_ in atherosclerosis. (A and D)** Typical immunofluorescence image of CTSK and integrin α_v_ in mouse aorta, respectively. **(B and E)** IHC images of CTSK and integrin α_v_ in mouse aorta, respectively (Designated regions indicated by red square frames in Figures are enlarged to show the details). **(C and F)** Quantitative IHC analysis of CTSK and integrin α_v_ positive area percentage, respectively (*n* = 3). Ctrl: control; AS: atherosclerosis.

We first examined the expression of CTSK and integrin α_v_ in atherosclerosis mice (Figure 1). Extensive red fluorescence-labeled CTSK was detected in atherosclerotic plaques (Figure 1A). In addition, IHC results also indicated that the expression of CTSK in atherosclerotic plaques was significantly higher than that in the normal aorta (control groups) (Figure 1B-C). Similarly, the expression of integrin α_v_ in atherosclerotic plaques was also significantly higher than that in healthy mice (Figure 1D-F). Therefore, these results suggested that the integrin-targeted and CTSK-sensitive nanoparticles we designed were feasible for atherosclerosis treatment.

Furthermore, we use a classical partial carotid ligation model of normolipidemic mice to simulate the initial stage of atherosclerosis (Figure S1A). The less curvature of the aorta (LC) is an atheroprone region that encounters disturbed flow compared to the gather curvature (GC). The left carotid artery (LCA) ligation created disturbed flow in LCA and AA regions, but the untied RCA and thoracic aorta (TA) regions with a steady flow (Figure S1A). *En face* staining showed that the expression of CTSK in the LC region was higher than that of the GC region 21 days after the ligation (Figure S1B). Indeed, a higher-level expression of CTSK in LCA was observed compared with the RCA (Figure S1B). In addition, tissue section staining results confirmed that the expression of CTSK, α_v_, and β_3_ in VECs of the LCA and AA regions was significantly higher than that of RCA and TA regions (Figure S1C).

### Synthesis and characterization of the functional polymer

Figure S2A showed the synthesis scheme of PLGA-Pep-PEG. Firstly, a Michael addition reaction occurred between the CTSK-responsive peptide with the maleimide (MAL) groups of PLGA-MAL. Excessive peptides were removed by dialysis. Subsequently, the purified PLGA-Pep reacted with the N-hydroxysuccinimide (NHS) groups of PEG-NHS to form an amide bond. The PLGA-Pep-PEG was obtained by further purification. The fluorescence resonance energy transfer (FRET) strategy was employed to monitor the hydrolysis events of CTSK. The donor-acceptor pair 5-(2′-aminoethyl) amino naphthalene sulfonic acid (EDANS) and 4-[[4′-(N, N-dimethylamino)phenyl]diazenyl] benzoic acid (DABCYL) have excellent spectral overlap between the fluorescence emission of the former and the absorption of the latter, resulting in efficient energy transfer 28. When CTSK hydrolyzed the responsive peptide, the blue fluorescence emitted by EDANS can be detected.

Figure S2B showed the FT-IR spectrum of PLGA-Pep-PEG, PLGA-MAL, PEG-NHS, and CTSK responsive peptides, confirming the presence of amide linkage in the PLGA-Pep-PEG. Characteristic FT-IR absorption bands of peptide were observed at 1592 cm^-1^ (aromatic C=C bending) and 3304 cm^-1^ (amine N-H stretch). The C=O stretching vibration in the amide bond was monitored at 1647 cm^-1^, which is the characteristic absorption of the amide II band. The presence of PLGA can be seen in 2992 cm^-1^ and 2938 cm^-1^ peaks, which is the stretching vibration of alkyl C-H in PLGA. Moreover, the characteristic absorption of the C-O-C group and C=O group in PEG were observed at 1086 cm^-1^ and 1647 cm^-1^, respectively. The proton nuclear magnetic resonance (^1^H NMR) spectrum of PLGA-Pep-PEG was shown in Figure S2C. The peak (2H) at δ 6.67 was the characteristic peak of maleimide in PLGA. However, the peak disappeared after binding with the peptide, indicating a new substance was generated. After coupling PLGA-Pep with PEG-NHS, the proton signal from the NHS leaving group of PEG-NHS was disappeared. In addition, the spectrum of PLGA-Pep-PEG (Figure S2C) contained the CH_3_ (δ 1.50-1.65) and CH (δ 5.18-5.27) of lactic acid, CH_2_ (δ 4.79-4.85) of glycolic acid in PLGA, and CH_2_ (δ 3.58-3.70) of PEG. All of these results confirmed that PLGA-Pep-PEG was successfully synthesized.

Figure S3A showed the synthesis scheme of PLGA-PEG-c(RGDfC). Similarly, the sulfhydryl group of the c(RGDfC) peptide reacted with the maleimide group of PLGA-PEG-MAL, forming PLGA-PEG-c(RGDfC). Excess c(RGDfC) peptides were removed by dialysis. Then, FT-IR and ^1^H NMR were used to characterize the structure of PLGA-PEG-c(RGDfC). As shown in Figure S3B, FT-IR spectra of the pure PLGA-PEG-c(RGDfC) confirmed that the PLGA-PEG-c(RGDfC) composite preserved each component's characteristic peaks. The peak that appeared at 1760.7 cm^-1^ was attributed to the stretching of carbonyl groups from the polymer. The peak around 1094.4 cm^-1^ belonged to the stretch band of the C=O bond. Peaks at 1660.8 cm^-1^ and 1547.6 cm^-1^ were assigned to C=O stretching and N-H bending vibration of amide, respectively. These results suggested a stable amide bond formed in PLGA-PEG-c(RGDfC). Furthermore, the ^1^H NMR spectrum showed that the characteristic peak of MAL disappeared due to the reaction of PLGA-PEG-MAL with c(RGDfC) (Figure S3C). All the results indicated the successful synthesis of PLGA-PEG-c(RGDfC).

As shown in Table S1, the Mn (number average molecular weight) and Mw (weight average molecular weight) of PLGA-Pep-PEG were 8.01 kDa and 13.65 kDa, respectively. The Mn and Mw of PLGA-PEG-c(RGDfC) were 7.16 kDa and 14.12 kDa, respectively. The theoretical molecular weight of PLGA-Pep-PEG and PLGA-PEP-c(RGDfC) was 8.5kDa and 7.5 kDa, respectively. The difference (<10%) between the theoretical molecular weight and the measured relative molecular weight may be caused by the difference in molecular structure between the standard and the synthesized polymer.

### Preparation and characterization of T/R NPs

**Figure 2:**
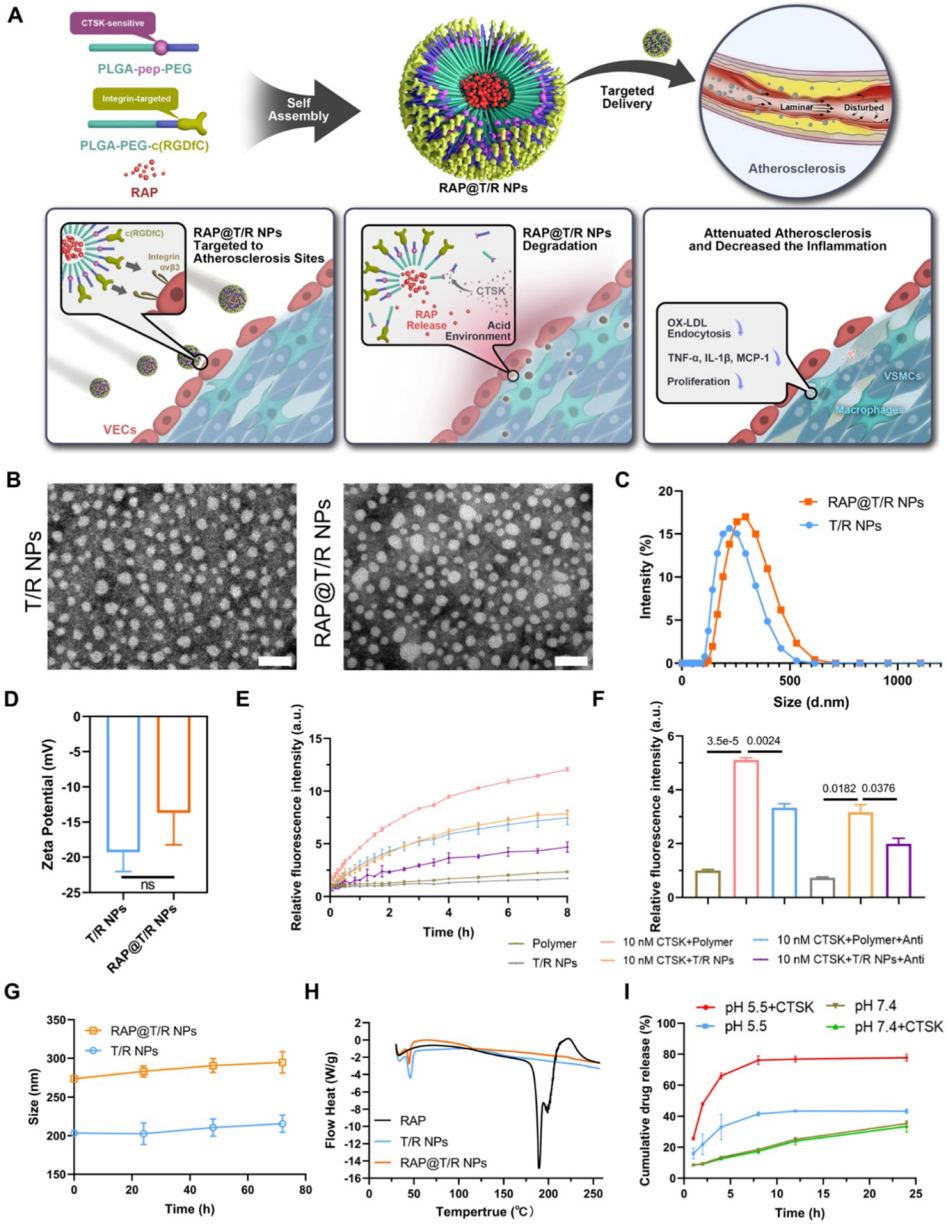
** Schematic of engineering of CTSK sensitive NPs and targeted therapy of atherosclerosis and characterization of nanoparticles. (A)** Schematic diagram of the components of the RAP@T/R NPs and targeted delivery RAP to treat atherosclerosis in response to CTSK. **(B)** Typical TEM images of T/R NPs and RAP@T/R NPs (Scale bar: 500 nm). **(C)** Size distribution profile and zeta potential **(D)** of T/R NPs and RAP@T/R NPs were measured using DLS analysis (*n* = 3). **(E)** The fluorescence intensity profile of PLGA-Pep-PEG and T/R NPs being incubated with or without CTSK enzyme *in vitro* at pH 5.5 (Polymer: PLGA-Pep-PEG, Anti: CTSK antibody). **(F)** PLGA-Pep-PEG and T/R NPs incubated with CTSK enzyme for 8 hours increase the fluorescence intensity absorption (*n* = 3). **(G)** Variation of size of RAP@T/R NPs in FBS 10% during 72 hours (*n* = 3). **(H)** DSC spectrum of free RAP, T/R NPs, and RAP@T/R NPs. (I) *In vitro* drug release of RAP@T/R NPs in the presence or absence of CTSK enzyme (*n* = 3).

The T/R NPs were composed of CTSK responsive polymers PLGA-Pep-PEG and targeting polymers PLGA-PEG-c(RGDfC), which self-assemble to form nanostructures. We hypothesized that the T/R NPs could target and bind to atherosclerotic lesions and release RAP in the presence of CTSK (Figure 2A).

As shown in Table S2, the DLC and DEE of different feeding ratios were quantitatively analyzed by HPLC. When the mass ratio of PLGA-Pep-PEG, PLGA-PEG-c(RGDfC) and the RAP was 2:2:1, the DLC and DEE were higher, reaching 14.62 ± 1.23% and 58.49 ± 4.94%, respectively. All subsequent experiments were performed with this feed ratio. The morphology of T/R NPs and RAP@T/R NPs was characterized by TEM. As shown in Figure 2B, the prepared NPs dispersed uniformly and exhibited a nearly spherical shape. DLS analysis indicated that T/R NPs and RAP@T/R NPs aggregated to particles with average sizes of 205.5 nm and 274.9 nm, respectively (Figure 2C). Meanwhile, the zeta potential of T/R NPs and RAP@T/R NPs were -19.2 ± 2.7 mV and -13.7 ± 4.6 mV, respectively (Figure 2D). The higher surface charges of T/R NPs and RAP@T/R NPs indicated that the nanoparticles have good colloidal stability.

Then, we employed FRET technology to evaluate the *in vitro* fluorescence activation of T/R NPs and PLGA-Pep-PEG polymer in the presence or absence of the CTSK enzyme. The fluorescence intensity gradually increased with the prolongation of incubation time with CTSK (Figure 2E). After 8 hours of incubation, the fluorescence intensity of the PLGA-Pep-PEG polymer and T/R NPs treatment with 10 nM CTSK was 5.12 ± 0.13 and 4.50 ± 0.10 folds higher respectively than that of the PLGA-Pep-PEG and T/R NPs without the CTSK treatment group, respectively (Figure 2F). Furthermore, the fluorescence absorbance intensity was significantly decreased when we added the CTSK antibody to the PLGA-Pep-PEG polymer or T/R NPs groups (Figure 2F). These results further confirmed that CTSK could specifically degrade PLGA-Pep-PEG polymers and T/R NPs. Subsequently, we evaluated the particles stability of T/R NPs *in vitro*. As shown in Figure 2G, there were no noticeable size changes of T/R NPs and RAP@T/R NPs in 10% FBS for 72 hours. DSC results showed that RAP has a high endotherm of approximately 190 °C, which is the melting point of RAP (Figure 2H). However, there was no melting point or glass transition absorption peak related to the monomolecular adsorption of drugs on the surface of the T/R NPs, which strongly proved that the RAP inside the T/R NPs is amorphous.

To evaluate the cumulative drug release profile of RAP, RAP@T/R NPs were transferred to dialysis bags and immersed in pH 7.4 and pH 5.5 buffer solution to simulate the physiological environment. In RAP@T/R NPs, the RAP release in pH 5.5 buffer solution (reached 41.6%) was faster than pH 7.4 buffer (18.6%) within 8 hours (Figure 2I). These results indicated that RAP@T/R NPs could respond to the weakly acidic microenvironment and accelerate RAP release. To ascertain the contribution of CTSK to drug release, the addition of 10 nM CTSK enzyme was added to the buffer system. The drug release from T/R NPs in pH 5.5 buffer solution plus CTSK, reaching 77.7%, was significantly higher than in pH 7.4 buffer solution plus CTSK (33.3%). These results further confirmed that the optimal activity of CTSK required an acidic environment. The rapid release of RAP from T/R NPs may result from CTSK cutting off the connection between the hydrophilic chain and the hydrophobic chain of the PLGA-Pep-PEG, resulting in the cleavage of the NPs, as well as enhanced drug release.

### *In vitro* cytotoxicity and hemocompatibility of T/R NPs

VECs, VSMCs, and macrophages are the major cell types in atherosclerotic lesions and were selected to examine the cytotoxicity of T/R NPs *in vitro*. As shown in Figure S4A, incubating HUVECs and RAW264.7 cells had high viability after being treated with 500 μg/mL of T/R NPs for 24 hours, and T/R NPs at concentrations less than 300 μg/mL had low cytotoxicity in HASMCs. In addition, the hemocompatibility analysis of T/R NPs showed no obvious hemolysis following the treatment with different concentrations of T/R NPs. Even with the treatment of 500 μg/mL of T/R NPs, the hemolytic percentage was only 2.2%, still below the criterion in the ASTM E2524-08 standard (5%) (Figure S4B). These results indicated that the T/R NPs did not cause RBCs damage and had excellent hemocompatibility.

### *In vitro* cellular uptake of T/R NPs

**Figure 3:**
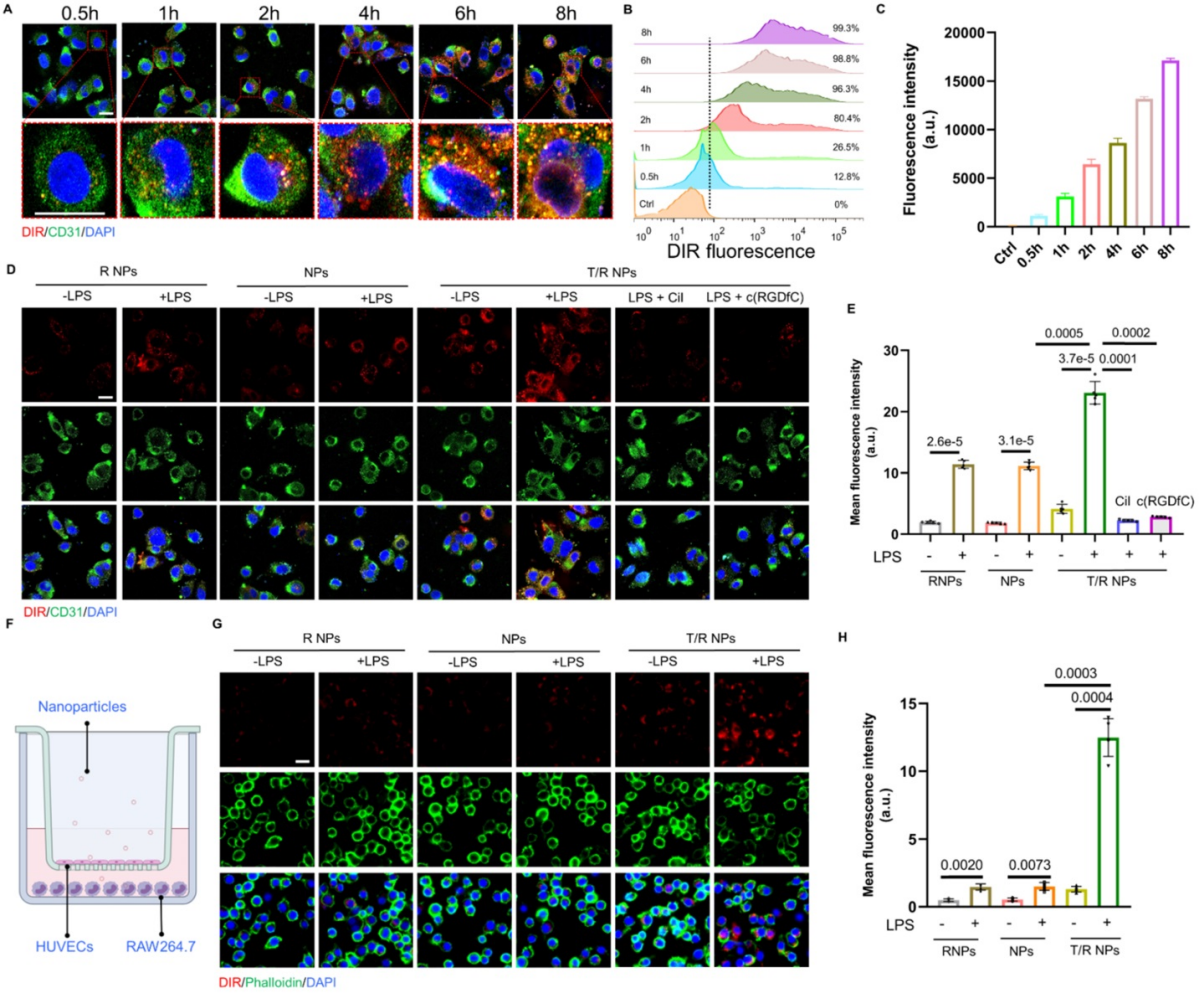
** Cellular uptake of T/R NPs in HUVECs and trans-endothelial transport capacity of T/R NPs. (A)** Confocal microscopy image of time-dependent cellular uptake of DIR@T/R NP (Scale bar: 20 µm). **(B)** Flow cytometry analysis of HUVECs uptake of DIR@T/R NPs. **(C)** Quantification of cellular uptake of DIR@T/R NPs in HUVECs at different time points (*n* = 3). **(D)** Cellular uptake of DIR@T/R NPs in HUVECs in the presence or absence of LPS as demonstrated by CLSM (Scale bar: 20 µm) and their mean fluorescence intensity **(E)** (*n* = 3). **(F)** Schematic illustration of the coculture model used. **(G)** Confocal microscopy images and mean fluorescence intensity **(H)** of DIR@T/R NPs in RAW264.7 cells in the bottom chamber (Scale bar: 20 µm) (*n* = 3).

To study the dynamic uptake of T/R NPs, we incubated DIR@T/R NPs with HUVECs and performed time-lapse observation using CLSM. The red fluorescent (DIR@T/R NPs) gradually enhanced intensities with the increased incubation duration (Figure 3A). We further quantified the cellular uptake of T/R NPs by using flow cytometry. Similarly, we found that the DIR positive cell number was 99.3% after 8 hours of incubation with DIR@T/R NPs, and the cell uptake profile was time-dependent (Figure 3B-C).

To further confirm the target binding capacity of the T/R NPs to integrins α_v_β_3_, HUVECs were pretreated with c(RGDfC) or cilengitide (a specific inhibitor of integrins α_v_β_3_ subunit), respectively. The c(RGDfC) peptide and cilengitide competitively bind to integrin α_v_β_3_, thereby reducing the cellular internalization of T/R NPs. As shown in Figure 3D-E, the fluorescence intensity of DIR in the LPS pretreated group was significantly higher than that in the control groups without LPS. In addition, the cellular uptake of DIR@T/R NPs was considerably increased compared with DIR@NPs and DIR@R NPs groups. Interestingly, the fluorescence intensity of DIR@T/R NPs was dramatically decreased in the groups pre-incubated with c(RGDfC) peptide and cilengitide compared with un-treated groups. These results confirmed that the T/R NPs could bind to integrins α_v_β_3_ receptors of inflammatory VECs.

Subsequently, the trans-endothelial efficiency of T/R NPs was detected by a trans-well model *in vitro*. The HUVECs and RAW264.7 cells were cultured in the upper and bottom chamber, respectively (Figure 3F). Under the condition of LPS stimulation, stronger red DIR fluorescence was detected in RAW264.7 cells than in control groups. Furthermore, more fluorescence intensity was detected in DIR@T/R NPs groups than that in DIR@NPs and DIR@R NPs groups. These results suggest that T/R NPs could effectively target and go through the inflammatory endothelial layers and accumulate in RAW264.7 cells (Figure 3G-H).

### Anti-atherosclerosis effects *in vitro*

**Figure 4:**
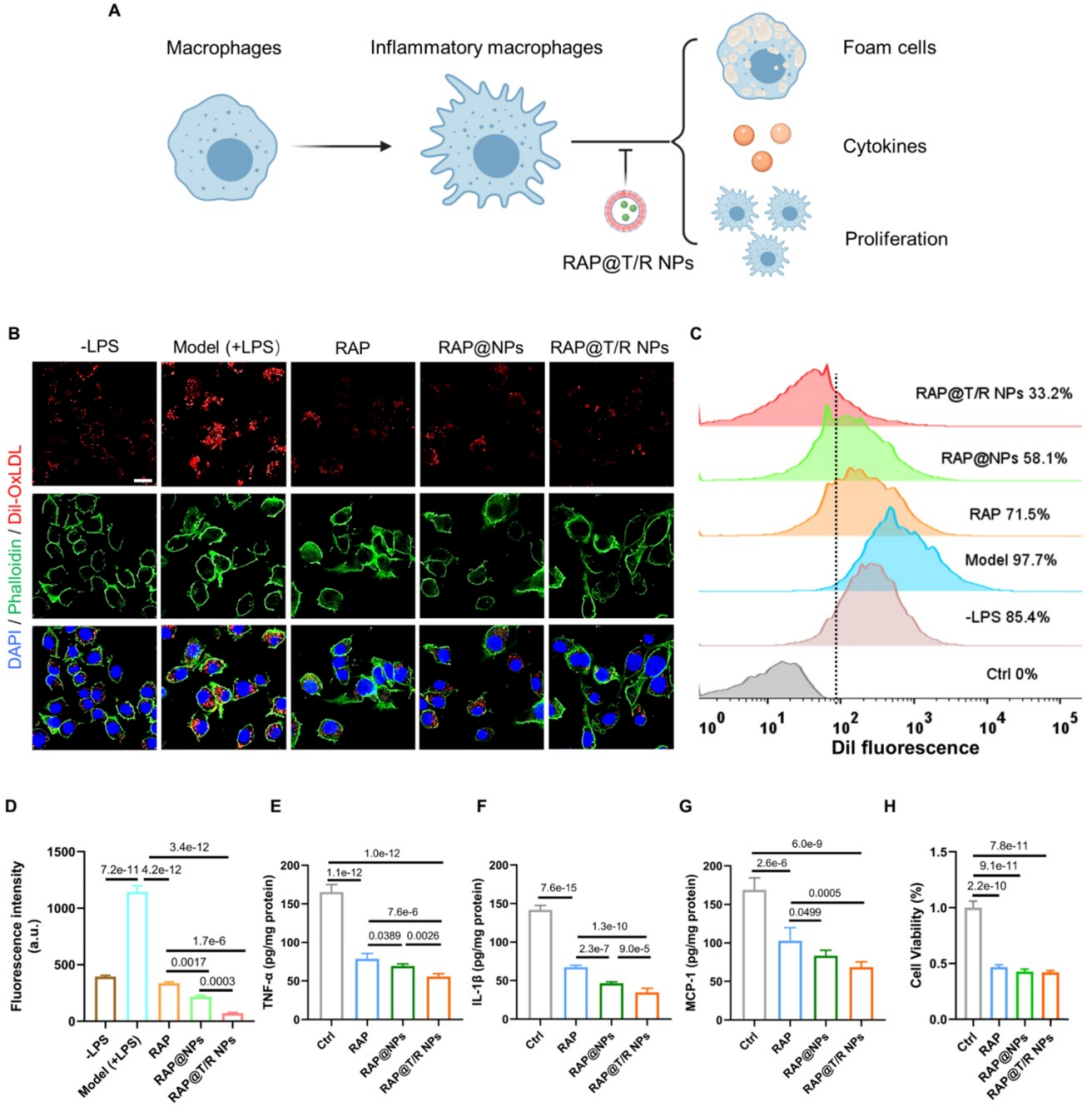
** The therapeutic effect of RAP@T/R NPs *in vitro*. (A)** RAP@T/R NPs Inhibit the formation of foam cells, releasing cytokines, and proliferation of macrophages. **(B)** RAP@T/R NPs inhibit phagocytosis of Dil-OxLDL by inflammatory macrophages (Scale bar: 20 µm). **(C)** Characterization of RAP@T/R NPs inhibits phagocytosis of Dil-OxLDL by inflammatory macrophages by flow cytometry (control groups: RAW264.7 cells without any treatment). **(D)** Quantification of uptake of Dil-OxLDL in inflammatory macrophages by flow cytometry. **(E-G)** ELISA detected typical inflammatory cytokines TNF-α (D), IL-1β (E), and MCP-1 (F) secreted by LPS treated RAW264.7 cells. **(H)** The cell viability of RAW264.7 cells after treatment RAP@T/R NPs. Data in (D-H) are mean ± SD (*n* = 3).

Macrophages residing in the intima are transformed into foam cells and release inflammatory factors mediated by the phagocytosis of low-density lipoprotein, which is an important event in atherogenesis 29. Accordingly, attenuation effects of RAP@T/R NPs on the internalization of Dil-labeled OxLDL were evaluated. Significantly red fluorescence intensity was found in LPS-treated groups compared with the control groups (Figure 4B). Furthermore, fluorescence images and flow cytometry results showed that the uptake of OxLDL by RAW264.7 cells was significantly decreased after free RAP and RAP@NPs treatment, and better effects were observed in RAP@T/R NPs treated groups (Figure 4C-D). Therefore, our results showed that the RAP@T/R NPs attenuated the formation of foam cells from macrophages by inhibiting the internalization of OxLDL.

The secretion of pro-inflammatory factors by inflammatory macrophages is thought to contribute to the development of atherosclerosis. We found that the secretion of pro-inflammatory factors TNF-α, IL-1β, and MCP-1 was significantly decreased after pre-incubation of 50 μg/mL of RAP@T/R NPs, 50 μg/mL of RAP@NPs or 5 μg/mL of free RAP (Figure 4E-G). Compared with free RAP and RAP@NPs treatment groups, RAW264.7 cells treated with the RAP@T/R NPs markedly reduced the levels of these inflammatory cytokines (Figure 4E-G). These findings verified that the RAP@T/R NPs could effectively attenuate inflammatory responses of macrophages *in vitro*. Combining with the previous results of resistance to foam cell formation, RAP@T/R NPs showed excellent anti-atherosclerosis ability and had a strong potential to inhibit inflammation *in vivo*.

Local proliferation of macrophages is a critical event in the genesis of the lesions of atherosclerosis 30-32. Therefore, we evaluated the effects of free RAP and RAP@T/R NPs on the proliferation of macrophages. As shown in Figure 4H, the proliferation of macrophages was significantly inhibited both in the RAP and RAP@T/R NPs treated groups compared with the control groups. However, there was no apparent difference between free RAP and RAP@T/R NPs, which might be due to the slow release of RAP from the T/R NPs *in vitro.*

### Acute toxicity of T/R NPs *in vivo*

Before evaluating the anti-atherosclerosis effects *in vivo*, the acute toxicity of T/R NPs was investigated (Figure S5). The routine blood examination showed that there were no significant differences in levels of RBCs, white blood cells (WBCs), platelets (PLT), lymphocyte, neutrophil granulocyte (GRAN), and hemoglobin (HGB) in mice between the control groups and T/R NPs treated groups. The biochemical blood indicator, including Alanine aminotransferase (ALT), alkaline phosphatase (ALP), albumin (ALB), γ-glutamyl transferase (γ-GT), creatinine (CREA), UREA, uric acid (UA), and total bile acid (TBA), maintained normal levels and showed no significant differences between the T/R NPs groups and NPs groups and the saline groups (Figure S5A). Furthermore, H&E staining results showed no noticeable pathological changes in the main organs, including the heart, liver, spleen, lung, and kidney (Figure S5B). These results indicated that the T/R NPs had good biocompatibility and were promising for atherosclerosis treatment.

### Blood circulation retention and targeting capability* in vivo*

**Figure 5:**
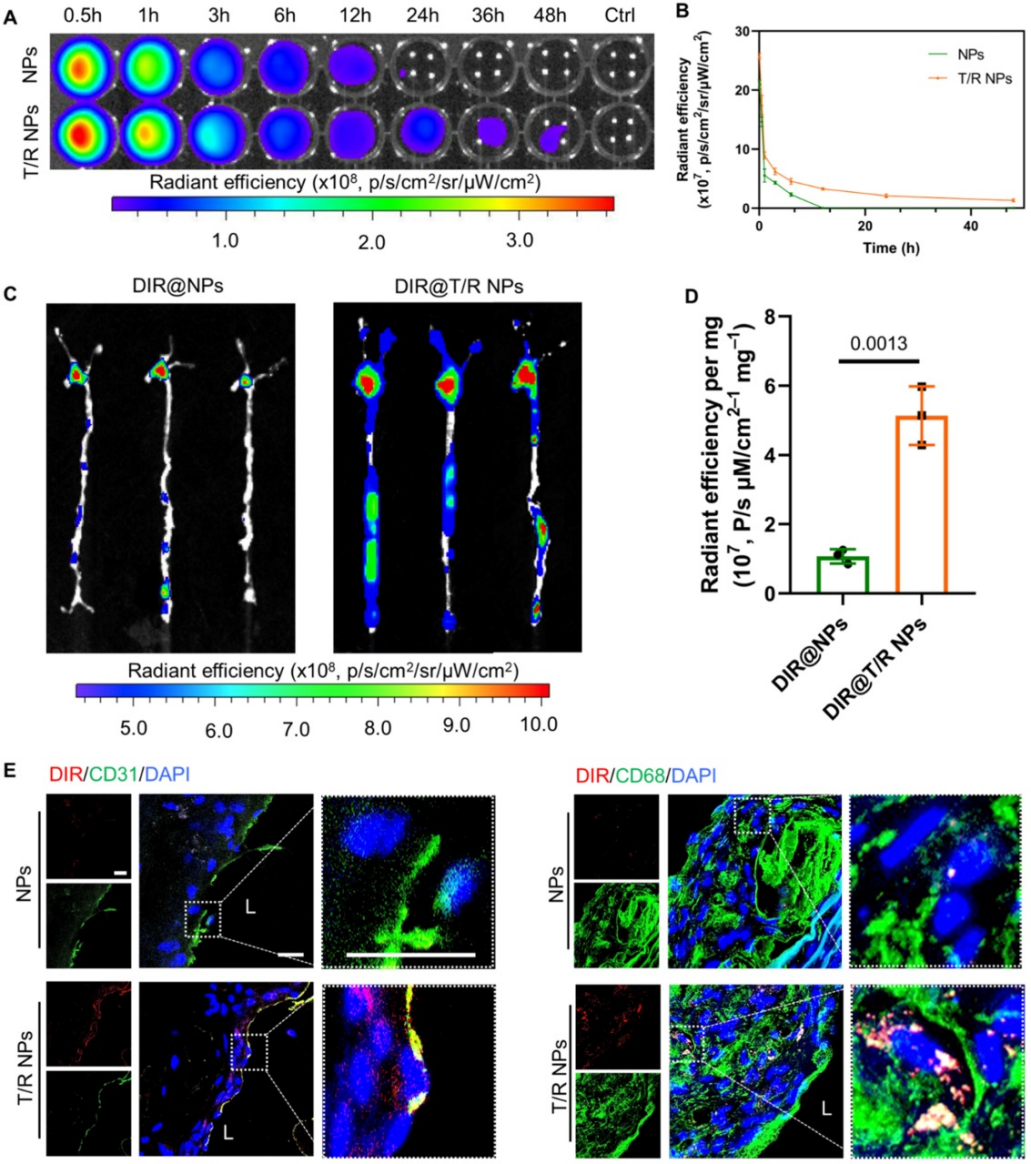
***In vivo* circulation and targeting capability of T/R NPs in mice after *i.v.* administration. (A)** *Ex vivo* image of whole blood collected at various time points after *i.v.* DIR@NPs and DIR@T/R NPs into C57BL/6 mice, and the fluorescence intensities quantification **(B)**. **(C)** *Ex vivo* fluorescence images and **(D)** quantitative analysis of DIR fluorescence in aorta 6 hours after DIR@NPs and DIR@T/R NPs injection into established atherosclerotic mice. **(E)** CLSM analysis of the co-location of DIR@T/R NPs (red) and endothelial cells (green), or DIR@T/R NPs (red) and macrophages (green) in atherosclerotic plaques. Data in (B and D) are mean ± SD (*n* = 3).

The *in vivo* pharmacokinetic profile of T/R NPs was first examined in C57BL/6 mice. The DIR@T/R NPs or DIR@NPs were intravenously injected through the tail vein, blood was collected at specific time points, and the fluorescence intensity was measured by the IVIS (Figure 5A). The results showed that the blood retention time of T/R NPs (48 h) was significantly longer than the NPs groups (Figure 5B). Thus, it is indicated that the T/R NPs experienced a long-time circulation in the blood.

Subsequently, we investigated the targeting ability of *i.v.* administration DIR@T/R NPs in ApoE^-/-^ mice were bearing atherosclerotic plaques. After injection for 6 hours, significant red fluorescence was observed in the AA and abdominal aorta, which is susceptible to atherosclerosis generation (Figure 5C). The average fluorescence intensities of the aorta in the DIR@T/R NPs administration groups were significantly higher than that DIR@NPs administration groups and control groups (Figure 5D). Furthermore, the fluorescence accumulated in main organs, including the heart, liver, spleen, lung, and kidney, as shown in Figure S6A. The amount of T/R NPs in the liver was lower than that NPs treatment groups (Figure S6B). The fluorescence images of cryosections from aorta plaque lesions showed the co-localization of T/R NPs with VECs and macrophages (Figure 5E). However, compared with the T/R NPs group, the DIR@NPs signal in the plaques was significantly lower, and no co-localization of NPs with VECs or macrophages was observed. These results demonstrated that the T/R NPs could specifically target and bind to the VECs and macrophages in the atherosclerotic plaques.

Similarly, we also evaluated the targeting capability of T/R NPs in the initial stage of atherosclerosis by using the partial carotid ligation mice. Because the expression of integrins α_v_β_3_ was highly in the AA and LCA of partial carotid ligation mice, the adsorption of DIR@T/R NPs in the aorta was significantly higher than that DIR@NPs treatment groups (Figure S7A-B). These results indicated that T/R NPs successfully target activated VECs at an initial stage of atherosclerosis development.

### Therapeutic efficacy in atherosclerotic mouse

**Figure 6:**
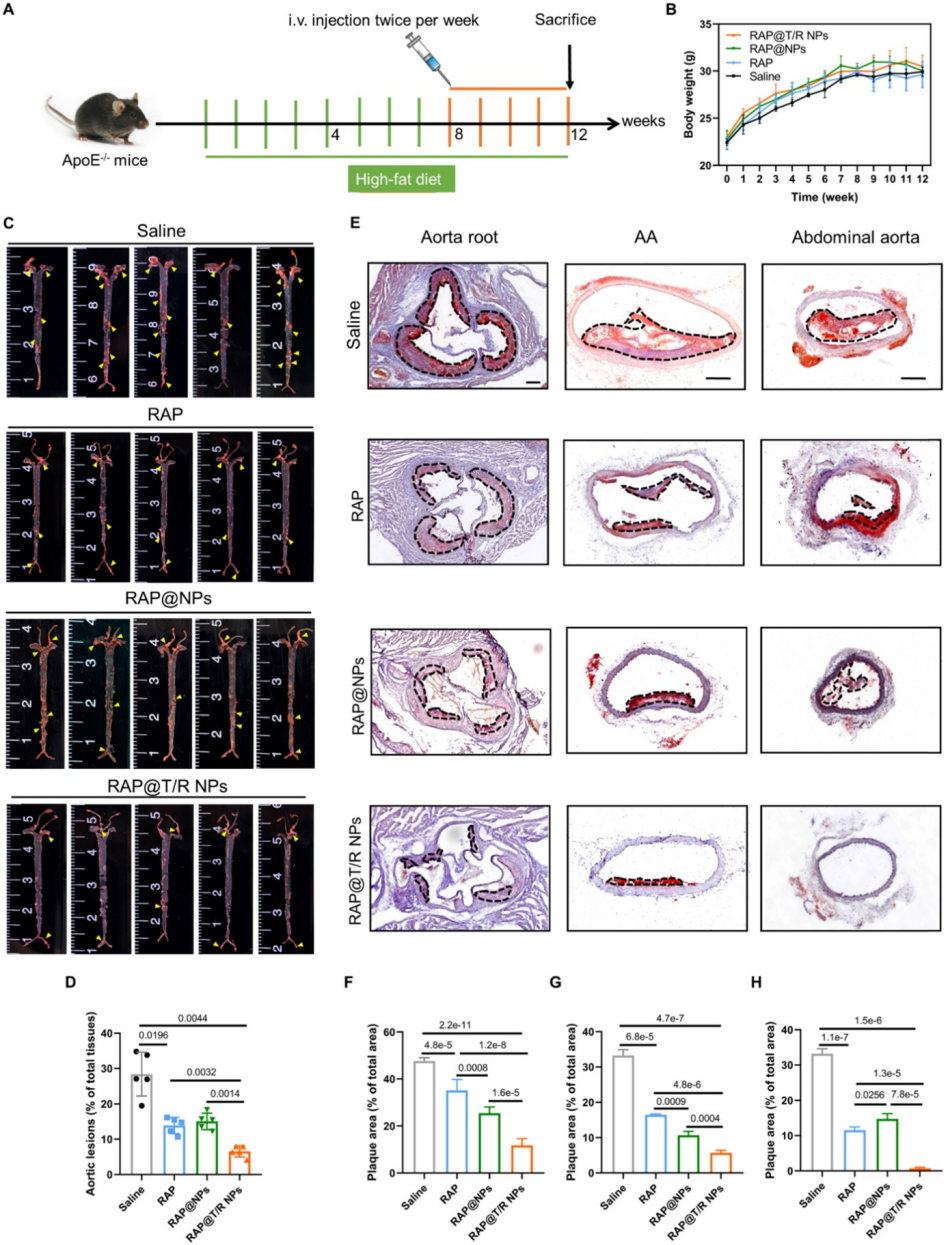
** Therapeutic effects of *i.v.* administration of RAP@T/R NPs in ApoE^-/-^ mice. (A)** Schematic illustration of the treatment protocols. **(B)** The body weight changes in ApoE^-/-^ mice were treated with various formulations (*n* = 15). **(C)** *En face* ORO-staining of aortas from ApoE^-/-^ mice after treatment with different formulations (saline, RAP, RAP@NPs, RAP@T/R NPs at a dose of 0.5 mg/kg RAP twice a week, *n* = 5, plaque lesions: yellow arrowheads). **(D)** Quantitative analysis of lesion area in aorta tissues (*n* = 5). **(E)** ORO-stained cryosections of the aortic root, AA, and abdominal aorta (plaque lesions: dotted frame, Scale bar: 200 µm, *n* = 5). **(F-H)** Quantitative analysis of the relative plaque area in sections of the aortic root (F), AA (G), and abdominal aorta (H) (*n* = 4-5).

Based on the above promising results, the therapeutic effect of RAP@T/R NPs was evaluated in a pathological atherosclerosis model in ApoE^-/-^ mice, following the treatment protocol shown in Figure 6A. After 12 weeks of high-fat feeding, the average weight of mice in each treatment group increased from about 22 grams to 31 grams, and there was no significant difference among the groups treated with RAP@T/R NPs, RAP@NPs, free RAP, and saline (Figure 6B). The whole aorta was harvested at the end of treatment, and *en face* was stained with ORO. As shown in Figure 6C, the lesion area ratio in the total aorta area was highest at 28.43% in the saline-treated groups. The RAP@NPs and free RAP moderately decreases the plaque area to 15.05% and 13.88%, respectively. Notably, the RAP@T/R NPs-treated groups significantly reduced the lesion area ratio to 6.56% of the total aorta area (Figure 6D). Subsequently, we conducted the ORO staining with cross-sections of prevalent atherosclerosis sites, including aorta root, AA, and abdominal aorta. As shown in Figure 6E, both free RAP, RAP@NPs, and RAP@T/R NPs could significantly reduce the deposition of lipids on the blood vessel and reduce the rate of vascular stenosis. After RAP@T/R NPs administration, the average area ratio of plaque to the vascular lumen was decreased from 47.67% to 11.77%, 33.29% to 5.68%, and 33.19% to 0.72% in the aorta root, AA, and abdominal aorta of the saline treatment groups, respectively (Figure 6F-H). These results revealed that the RAP@T/R NPs could effectively attenuate the progression of atherosclerosis.

**Figure 7:**
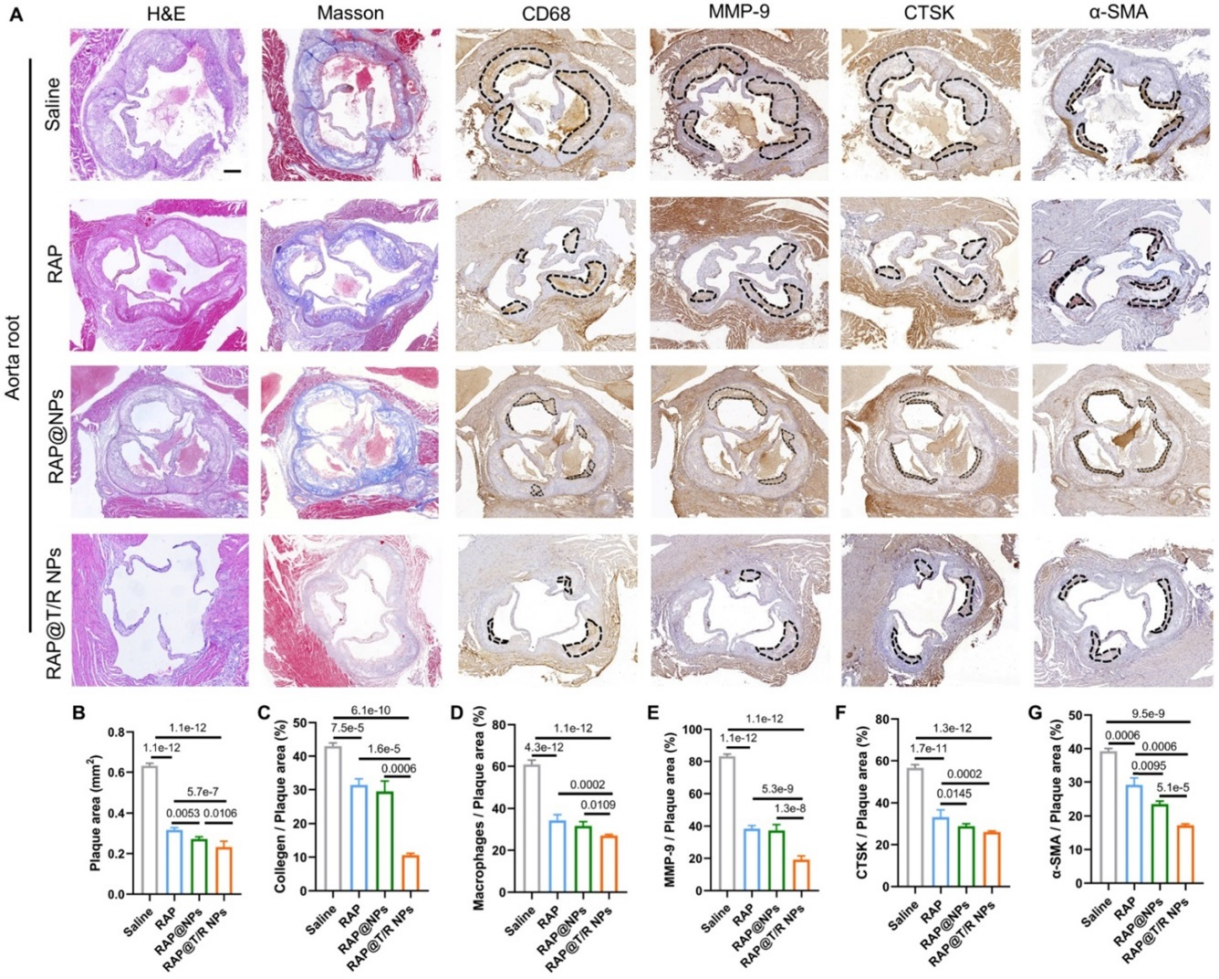
** Histochemistry analysis of aortic root sections from ApoE^-/-^ mice after different treatments. (A)** Representative photographs of aortic root sections from ApoE^-/-^ mice after treatment with different formulations (saline, RAP, RAP@NPs, RAP@T/R NPs at a dose of 0.5 mg/kg RAP twice a week) stained with H&E, Masson's trichrome, antibody to CD68, antibody to MMP-9, CTSK, and antibody to α-SMA (positive arear: dotted frame, Scale bar: 200 µm, *n* = 5). **(B-G)** Quantitative analysis of plaque area (B), collagen area relative to plaque area (C), macrophage area relative to plaque area (D), MMP-9 area relative to plaque area (E), CTSK area relative to plaque area (F), and VSMCs area relative to plaque area (G) (*n* = 5).

Next, histological and immunohistochemical analysis was conducted to detect the composition of aorta root, AA, TA, abdominal aorta, and brachiocephalic artery, respectively. As shown in Figure 7A, H&E staining on the aortic root showed many large plaques and necrotic cores in the saline groups. However, the plaque areas were significantly reduced in both free RAP and RAP@T/R NPs treatment groups. Especially, the RAP@T/R NPs groups showed better therapeutic effects than free RAP treatment groups (Figure 7A-B). The macrophages in the necrotic nucleus are mainly from the infiltration of monocytes in circulation, which are positively related to plaque development and disease severity 33. In addition, the expression of MMP-9 and CTSK was positively correlated with plaque vulnerability 34, 35. Therefore, we separated staining with CD68, MMP-9, and CTSK antibodies. Results indicated that RAP@T/R NPs effectively decreased the number of macrophages and the expression of MMP-9 and CTSK in the plaque area (Figure 7A and C-E). These results demonstrated that RAP@T/R NPs could prevent the infiltration of macrophages and maintain the stability of plaques. Furthermore, Masson's trichrome staining detected the collagen content around plaques. RAP@T/R NPs significantly decreased collagen content (Figure 7A-F) compared with other groups. IHC analysis for α-smooth muscle actin (α-SMA, SMCs marker) showed that the number of SMCs dramatically decreased in the aortic root plaques, particularly in the RAP@T/R NPs-treated groups. These findings confirmed that RAP delivered by T/R NPs could more effectively inhibit the proliferation of SMCs in the progression of atherosclerosis.

**Figure 8:**
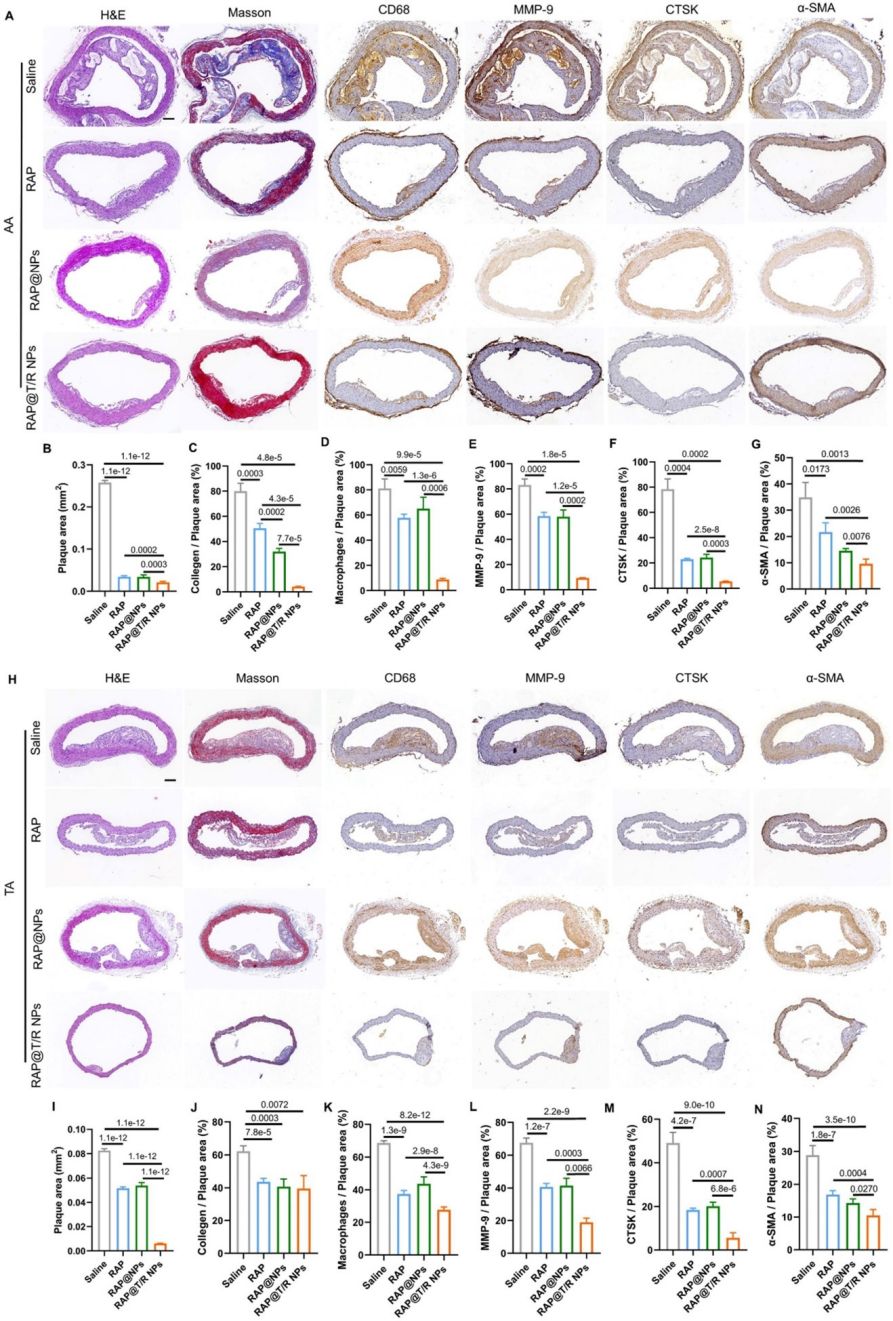
** Histochemistry analysis of AA and TA sections from ApoE^-/-^ mice after different treatments. (A and H)** Representative photographs of AA and TA sections from ApoE^-/-^ mice after treatment with different formulations (saline, RAP, RAP@NPs, RAP@T/R NPs at a dose of 0.5 mg/kg RAP twice a week) stained with H&E, Masson's trichrome, antibody to CD68, antibody to MMP-9, CTSK, and antibody to α-SMA (Scale bar: 100 µm). **(B-G, I-N)** Quantitative analysis of plaque area (B and I), collagen area relative to plaque area (C and J), positive macrophage area relative to plaque area (D and K), MMP-9 area relative to plaque area (E and L), CTSK area relative to plaque area (F and M), and VSMCs area relative to plaque area (G and N) in AA and TA. Data in (B-G, I-N) are mean ± SD (*n* = 5).

Similarly, the extent of atherosclerosis of other aorta sites, including AA, TA, abdominal aorta, and brachiocephalic artery, were evaluated by H&E staining, Masson's trichrome staining, and IHC (MMP-9, CTSK, CD68, and α-SMA) (Figures 8A-N and S8A-N). These results indicated that the RAP@T/R NPs treatment reduced the number of SMCs, macrophage infiltration into atherosclerosis lesions, and the expression of MMP-9 and CTSK.

Compared with the RAP@NPs and free RAP treatment groups, major pro-inflammatory cytokines (TNF-α, IL-1β, and MCP-1) in aorta tissues and blood serums of ApoE^-/-^ mice were significantly decreased in the RAP@T/R NPs treatment groups (Figure S9A and B). These data supported that the RAP@T/R NPs could reduce systemic and local inflammation* in vivo*.

In addition, the pharmacokinetic profiles of RAP in the aorta after 4 weeks of treatment were measured by HPLC. As shown in Figure S10, the content of retained RAP in the aorta of the RAP@T/R NPs (36.6 ± 0.4 μg/g) treatment group was significantly higher than that of the free RAP (6.8 ± 0.7 μg/g) and RAP@NPs (12.6 ± 0.5 μg/g) treatment groups. This result confirms that T/R NPs could enhance the targeting and local bioavailability of RAP.

### Biosafety assessment

At the end of the 4 weeks treatment, we evaluated the potential side effects of T/R NPs. Our results showed that there were no significant differences in blood cells (RBCs, WBCs, PLT, lymphocytes, neutrophils) and biochemical blood indicators (ALT, ALP, ALB, γ-GT, CREA, UREA, UA, TBA) between RAP@T/R NPs, RAP@NPs, free RAP and saline groups (Figure S11A). Furthermore, the H&E staining results also demonstrated no noticeable pathological changes found in the main organs (heart, liver, spleen, lung, and kidney) after RAP@T/R NPs injection (Figure S11B).

## Discussion

Common anti-atherosclerosis medicines include cholesterol-lowering medications, antiplatelets, and vasodilators. However, these applications have some limitations for atherosclerosis therapy due to their non-specific distribution, poor water solubility 2, and rapid metabolism for clearance. In addition, long-term treatment can cause serious side effects, such as liver damage, muscle symptoms, and diabetes mellitus 3. Here, we reported a CTSK-sensitive nanoparticles drug delivery system that effectively targets atherosclerosis sites via αvβ3 and releases RAP in response to CTSK. The integration of integrins α_v_β_3_ targeting and CTSK responsive properties in nanoparticles enables a specific and compelling RAP release at the atherosclerotic sites, minimizing the side effects on other tissues and organs.

After intravenous injection, most nanoparticles may be recognized and eliminated by phagocytes in the blood. A practical method is to modify hydrophilic PEG on the surface of nanoparticles. Due to the flexibility and hydrophilicity of PEG, it can form a dense hydration layer on the nanoparticle's surface. This layer can prevent protein adsorption caused by steric repulsion, avoid the mononuclear phagocytes system recognition and clearance, prolong the residence time in the blood, and enhance targeted drug delivery 36, 37. Indeed, our results showed that the PEGylated T/R NPs had extended circulation time (48 hours), which further enhances nanoparticles targeting atherosclerotic sites to improve therapeutic efficiency. It is worth noting that biomimetic nano-drug delivery systems such as RBCs membrane 37, platelets membrane 38, 39, and macrophages membrane 40, 41 coating strategies have been employed to prolong blood circulation time and target atherosclerosis sites. However, several technological issues remain to be addressed before this technology can be developed for therapies, including uneven and incomplete coating, short shelf life, and limited scalability 42, 43.

Recently, active targeting strategies have been used for nanoparticle modification to enhance drug delivery efficiency 44, 45. The nanoparticles can be designed to bind to the specific cells or molecules in the atherosclerotic lesions through their particular ligands. For example, ICAM-1, VCAM-1, P-selectin, and integrins α_v_β_3_ of VECs, scavenger receptors (MSR-A, SR-BI, and CD36), and mannose receptors (CD206) on the surface of macrophages, and type IV collagen in plaque lesions have been used as targets for nanoparticles binding. Since integrins α_v_β_3_ are highly expressed in atherosclerosis lesions, various targeted strategies have been employed to treat vascular diseases based on the integrins α_v_β_3_ 9, 46, 47. Our results also demonstrated that integrin α_v_β_3_ is highly expressed in atherosclerotic lesions. Therefore, we used cyclic peptides c(RGDfC) modified nanoparticles to target integrins α_v_β_3_ of atherosclerosis and showed that the targeted nanoparticles effectively accumulated aggregated in atherosclerotic plaques. In addition, our study also confirmed that T/R NPs could cross the inflammatory VECs layer and be endocytosed by macrophages to exert a therapeutic effect. However, integrins are also highly expressed in many organs, such as the liver 48, 49, which may lead to increased distribution of RAP in the liver and potential toxicity in this organ, as observed in our study. Stimulus-responsive targeting strategies can make up for this deficiency. Enzyme-responsive drug delivery systems have been widely employed to diagnose and treat tumors 50. Still, there are few reports of enzyme-responsive drug delivery systems for the treatment of atherosclerosis. Therefore, we used CTSK-responsive nanoparticles loaded with RAP to suppress the non-specific RAP release and demonstrated no significant toxicity in other organs.

Considering other microenvironment changes at atherosclerosis sites, nanoparticles have been engineered for drug release in response to ROS 6, 7, 51 and low pH values 7, 9, but these changes are atherosclerotic tissue-specific. Atherosclerosis is accompanied by ECM remodeling, and plaques are rich in extracellular matrix-degrading enzymes such as MMPs 10 and cysteine proteases 11. Interestingly, CTSK only exposes its protease's active domain under acidic pH and thus can only exert its optimal activity under weakly acidic conditions 20. Therefore, T/R NPs can release RAP quickly in response to a high concentration of CTSK and an acidic microenvironment at atherosclerotic lesions but have a meager release rate in the liver and other organs under a normal physiological microenvironment. Such a particular CTSK responsive drug delivery strategy could avoid the side effects caused by the non-specific distributions of the nanoparticles. Overall, T/R NPs, with their microenvironment-responsive properties, enable the selective delivery of RAP to atherosclerosis sites and offer improved *in vivo* safety.

Besides CTSK, other members of the cathepsin family, including cathepsin B (CTSB) 52, cathepsin S (CTSS) 53, cathepsin L (CTSL) 54, and cathepsin C (CTSC) 55, also play a critical role in the development of atherosclerosis. Kenneth et al. previously reported that ApoE^-/-^ and CTSS mice deficiency fed with the high-fat diet had fewer acute plaque ruptures and smaller plaque sizes than ApoE^-/-^ mice 56. Furthermore, pharmacological CTSS inhibition of in ApoE^-/-^ mice also reduced elastic plate rupture and macrophage infiltration and the number of buried fibrous caps and plaque size 57. Therefore, other responsive nanoparticles designed based on the enzymatic reaction substrates of other cathepsin family members may be potentially employed for atherosclerosis treatment. In addition to atherosclerosis, CTSK is upregulated in other diseases, such as osteoporosis 58, cancer 59, 60, aortic aneurysms 61, and heart disease 62, and CTSK responsive strategy can be used to release drugs to treat these diseases 63, 64.

In conclusion, we have successfully engineered an integration of integrins α_v_β_3_ targeting and CTSK responsive nanoparticles and demonstrated promising potential for atherosclerosis treatment.

## Supplementary Material

Supplementary figures and tables.
