# Supplementary material for: Inflammatory endothelium-targeted and cathepsin responsive nanoparticles are effective against atherosclerosis
Source: Theranostics. 2022 May 16;12(9):4200–20. doi: 10.7150/thno.70896 (PMC9169363; doi:10.7150/thno.70896)
Supplement: Supplementary file 1 — Supplementary figures and tables. [file thnov12p4200s1.pdf]

Supplementary Information

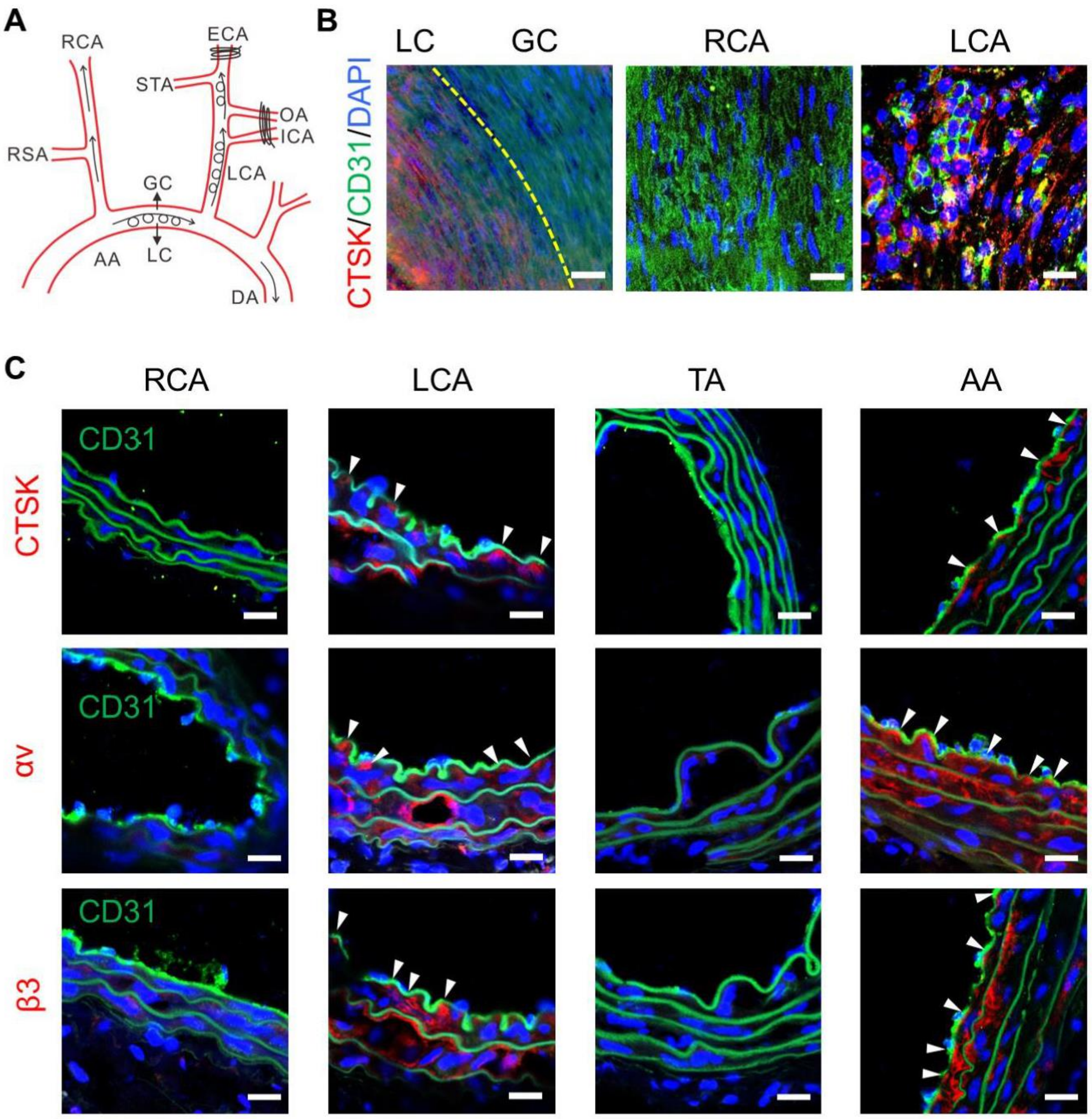

Figure S1. The expression of CTSK and integrins $\alpha_v\beta_3$ in the early stage of atherosclerosis. (A) The diagram of partial carotid artery ligation. Right subclavian artery (RSA), right carotid artery (RCA), external carotid artery (ECA), superior thyroid artery (STA), occipital artery (OA), internal carotid artery (ICA), left carotid artery (LCA), left subclavian artery (LSA), aortic arch (AA), descending aorta (DA), lesser (LC) or greater (GC) curvature regions of the aortic arch. (B) *En face* staining for CTSK (red) and CD31 (green) on the LC or GC and the RCA, LCA. Nuclei were stained with DAPI (Scale bar: 20 μm). (C) RCA, LCA, TA, AA tissues sections from wild-type mice 21 days after ligation were immunostained for CTSK (red; arrowheads), αv (red; arrowheads) or β3 (red; arrowheads) and counterstained with CD31. Nuclei were stained with DAPI (Scale bar: 20 μm).

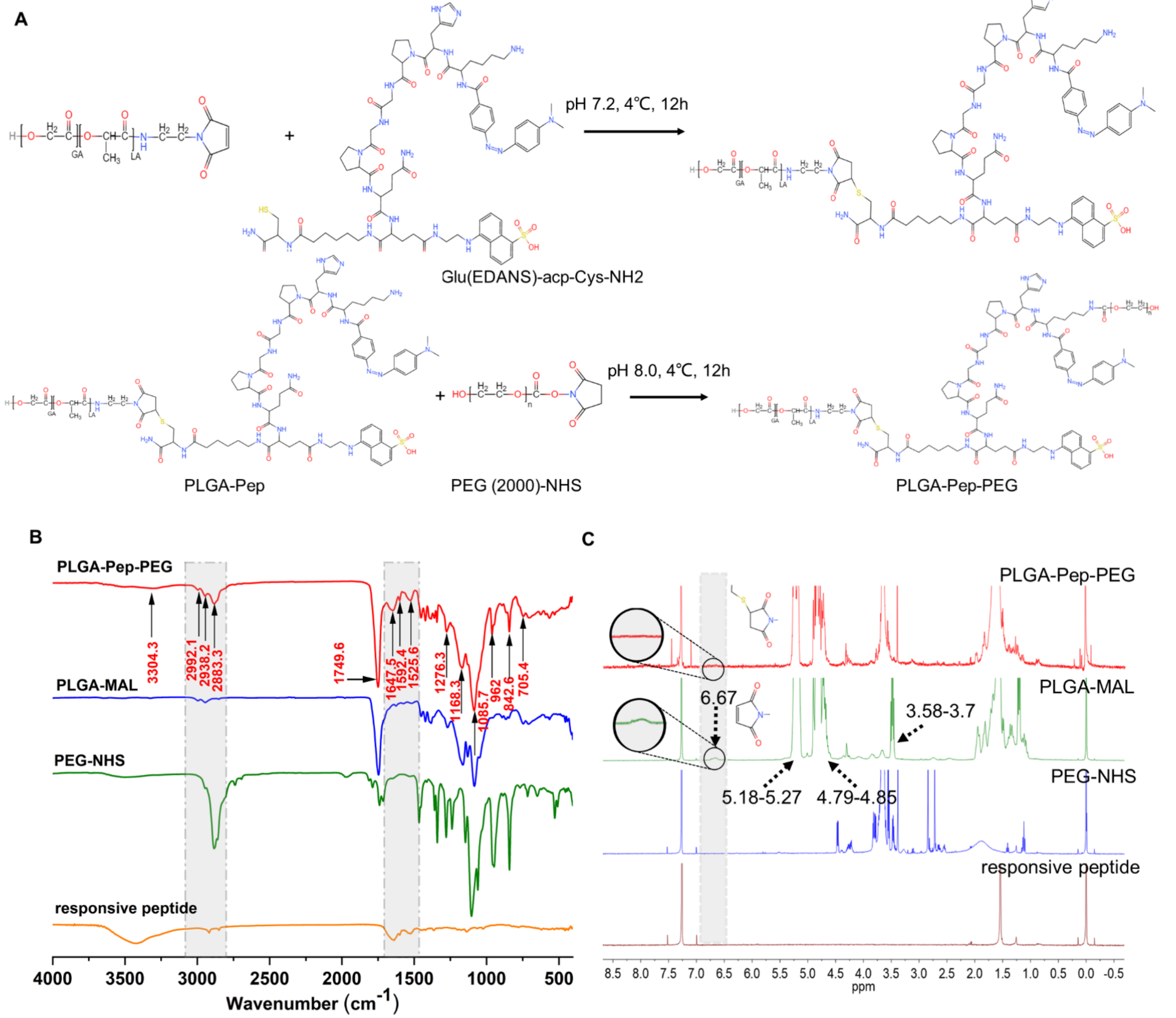

Figure S2. Synthesis route and structure characterization of PLGA-Pep-PEG. (A) A Synthesis route of PLGA-Pep-PEG. (B) FT-IR spectrum of PLGA-Pep-PEG. (C) ^{1}H NMR spectrum of PLGA-Pep-PEG.

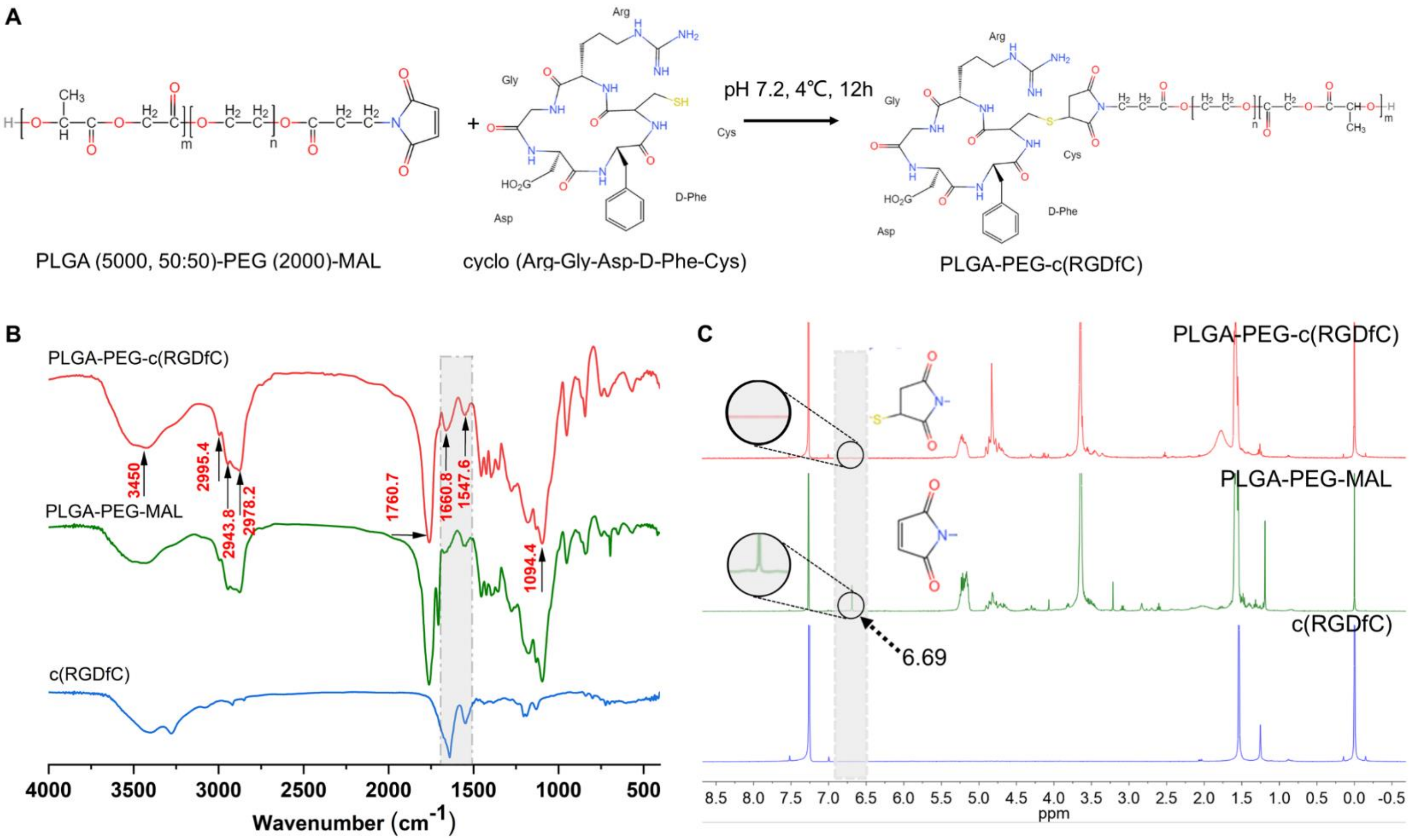

Figure S3. Synthesis route and structure characterization of PLGA-PEG-c(RGDfC). (A) A Synthesis route of PLGA-PEG-c(RGDfC). (B) FT-IR spectrum of PLGA-PEG-c(RGDfC). (C) ^{1}H NMR spectrum of PLGA-PEG-c(RGDfC).

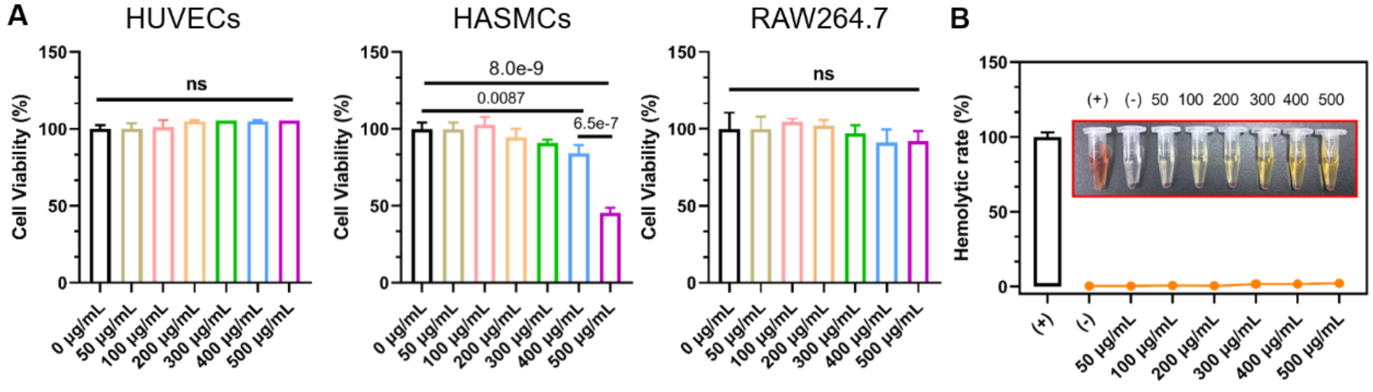

Figure S4 *In vitro* cytotoxicity and hemolytic evaluation of T/R NPs. (A) Cell viability values of HUVECs, HASMCs, and RAW264.7 cells after incubation with various doses of T/R NPs 24 hours. (B) Hemolysis analysis of blood samples after exposure to various doses of T/R NPs. Data in (A and B) are mean ± SD (n = 3).

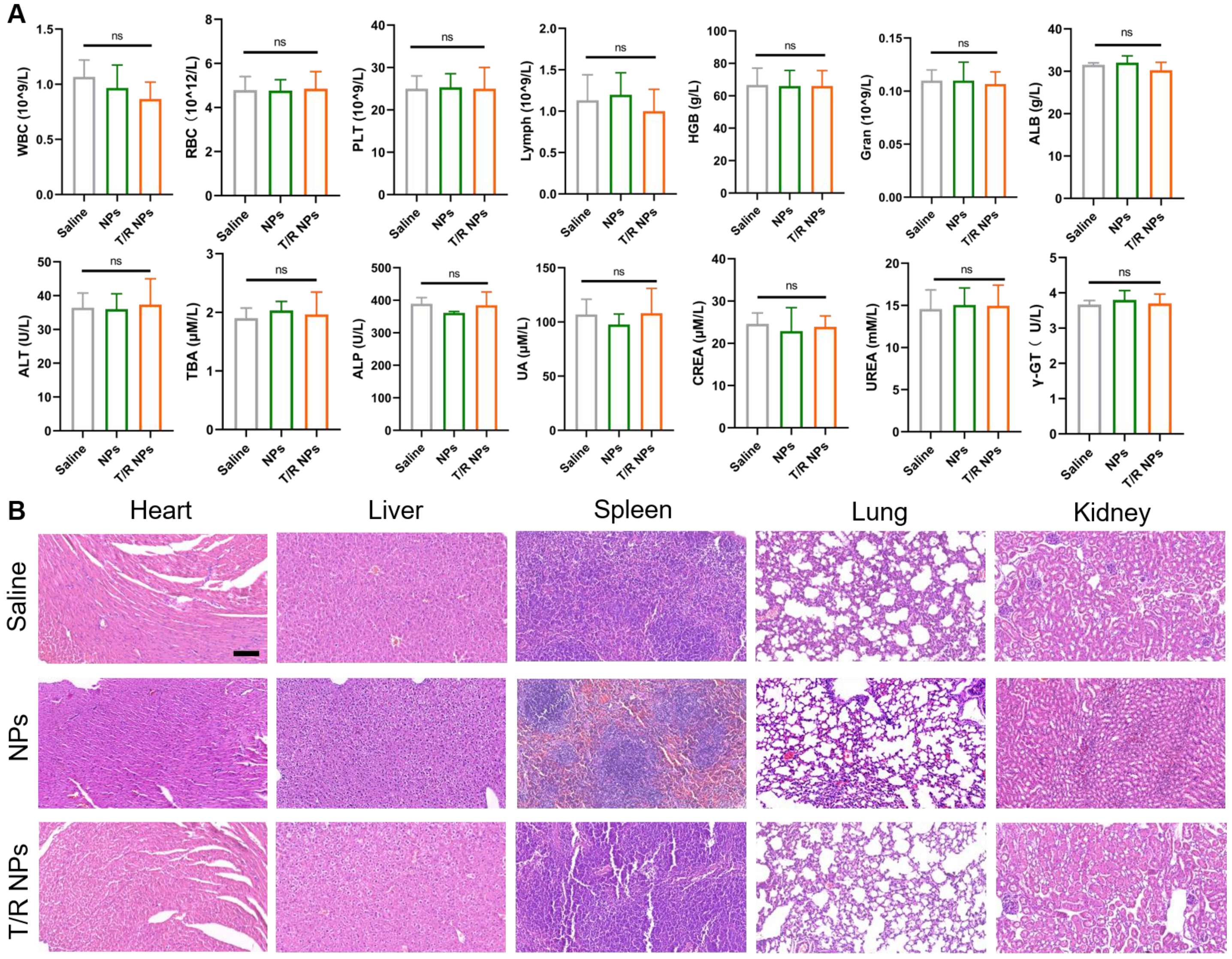

Figure S5. Toxicological evaluations and H&E staining after 24 hours of treatment. (A) Routine blood and biochemical assays. RBCs, WBCs, PLT, lymphocytes, GRAN, HGB, ALT, ALP, ALB,γ-GT, CREA, UREA, UA, and TBA. (B) H&E-stained images of main organs from mice (Scale bar: 100 μm). Data in (A) are mean ± SD (*n* = 3).

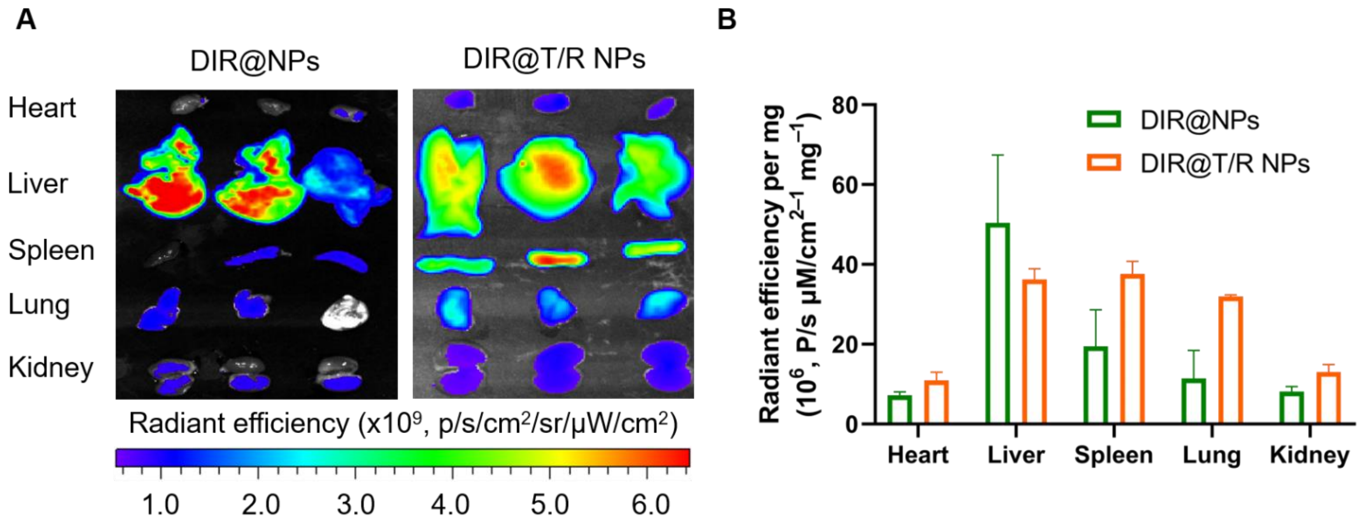

Figure S6. Distribution of DIR@T/R NPs and DIR@NPs in various organs after *i.v.* injection in mice. (A) *Ex vivo* fluorescence images and (B) quantitative analysis of DIR fluorescence in main organs 6 hours after DIR@NPs and DIR@T/R NPs injection into established atherosclerosis mice. Data in (B) are mean ± SD (n = 3).

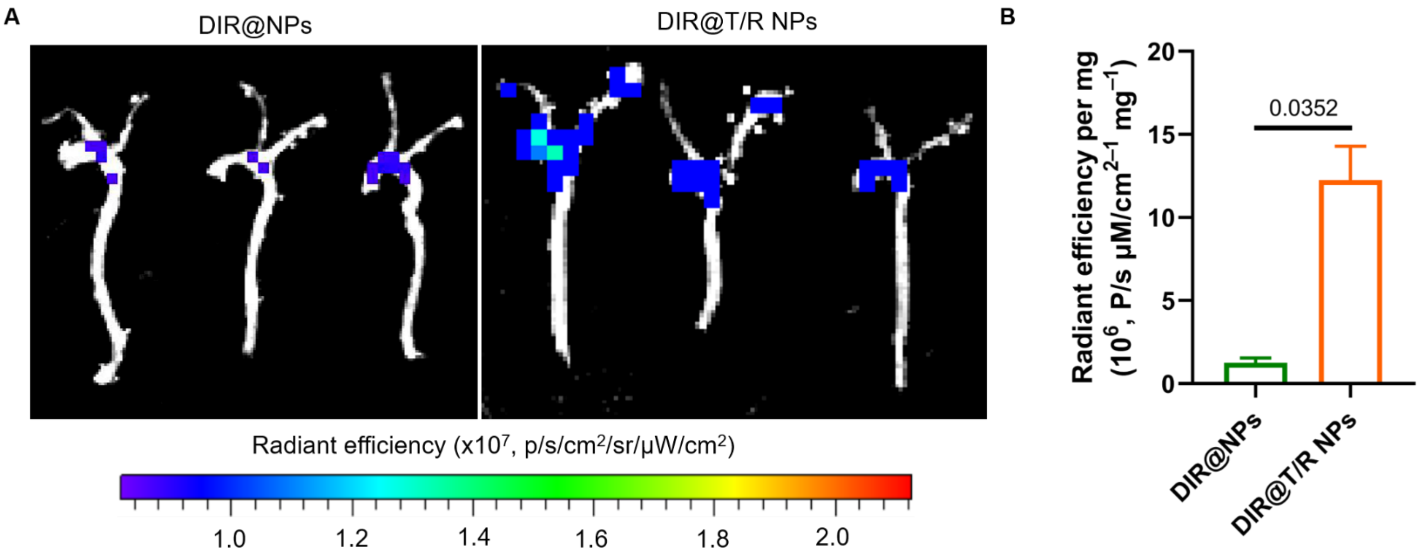

Figure S7. Targeting capability of T/R NPs in mice after ligation. (A) *Ex vivo* fluorescence images and (B) quantitative analysis of DIR fluorescence in aorta 6 hours after DIR@NPs and DIR@T/R NPs injection into partial carotid ligation mice. Data in (B) are mean ± SD (n = 3).

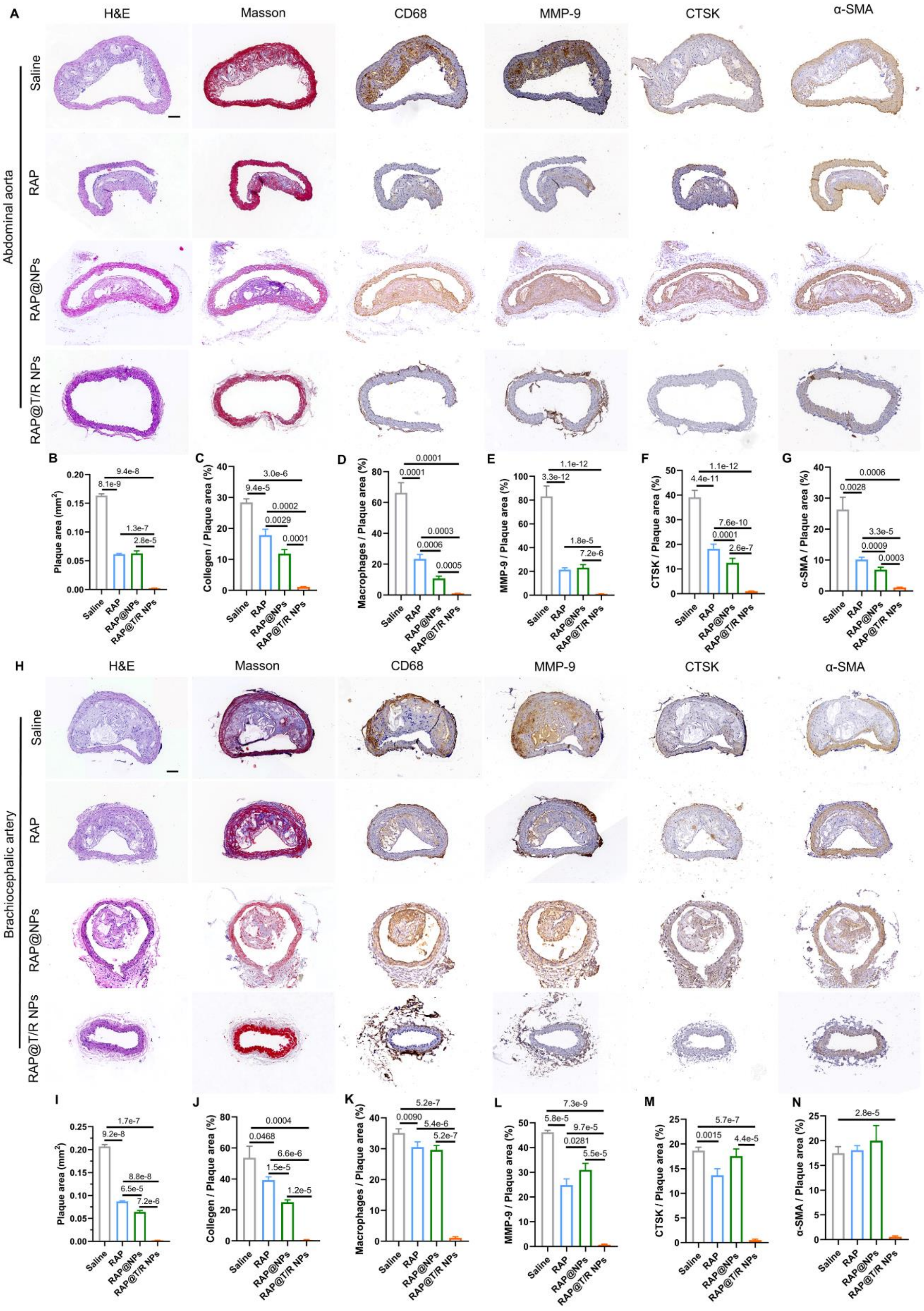

Figure S8. Histochemistry analyses the abdominal aorta and brachiocephalic artery sections from ApoE$^{-/-}$ mice after different treatments. (A and H) Representative photographs of the abdominal aorta and brachiocephalic artery sections stained with H&E, Masson's trichrome, antibody to CD68, antibody to MMP-9, CTSK, and antibody to α-SMA (Scale bar: 100μm). (B-G, I-N) Quantitative analysis of plaque area (B and I), collagen area relative to plaque area (C and J), positive macrophage area relative to plaque area (D and K), MMP-9 area relative to plaque area (E and L), CTSK area relative to plaque area (F and M), and VSMCs area relative to plaque area (G and N) in the abdominal aorta and brachiocephalic artery. Data in (B-G, I-N) are mean ± SD (*n* = 3).

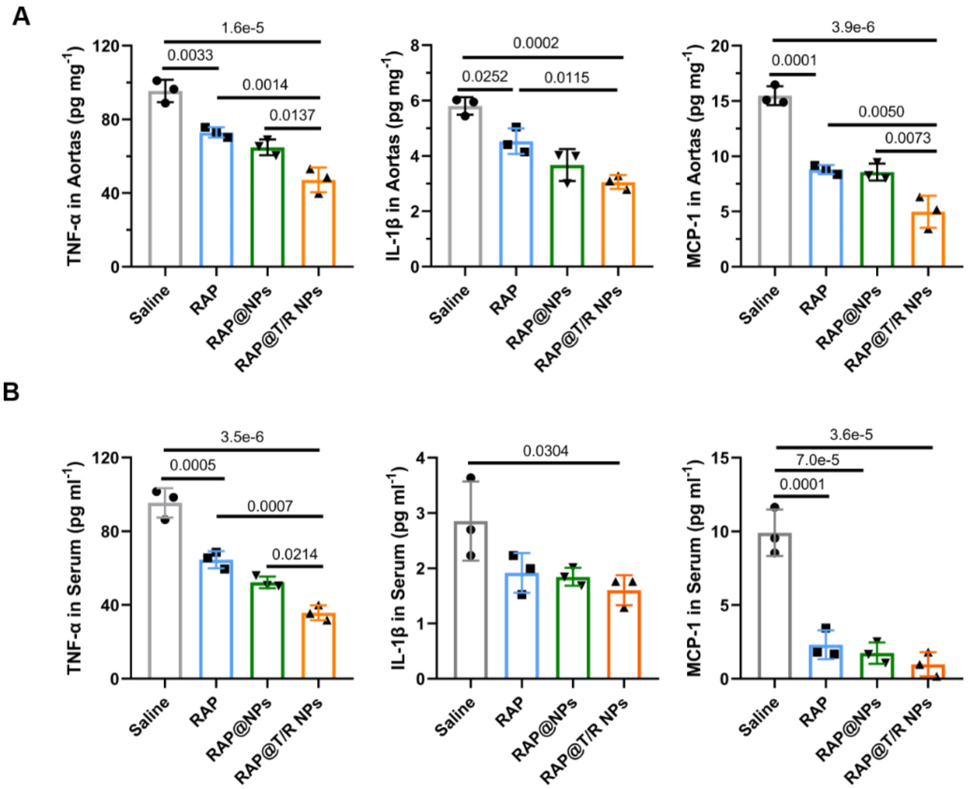

Figure S9. Inhibition of systemic inflammation and local inflammation in ApoE$^{-/-}$ mice by RAP@T/R NPs treatment. (A and B) The levels of inflammatory cytokines TNF-α, IL-1β, and MCP-1 in aortas and serum were collected from atherosclerotic mice after treatment with various formulations (saline, RAP, RAP@NPs, RAP@T/R NPs at a dose of 0.5 mg/kg RAP twice a week). Data in (A and B) are mean ± SD (*n* = 3).

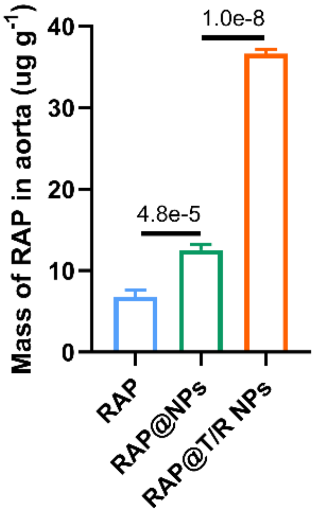

Figure S10. The mass of RAP in the aorta. The content of RAP in aortas was collected from atherosclerotic mice after 4 weeks of treatment with various formulations (RAP, RAP@NPs, RAP@T/R NPs at a dose of 0.5 mg/kg RAP twice a week, *n* = 3).

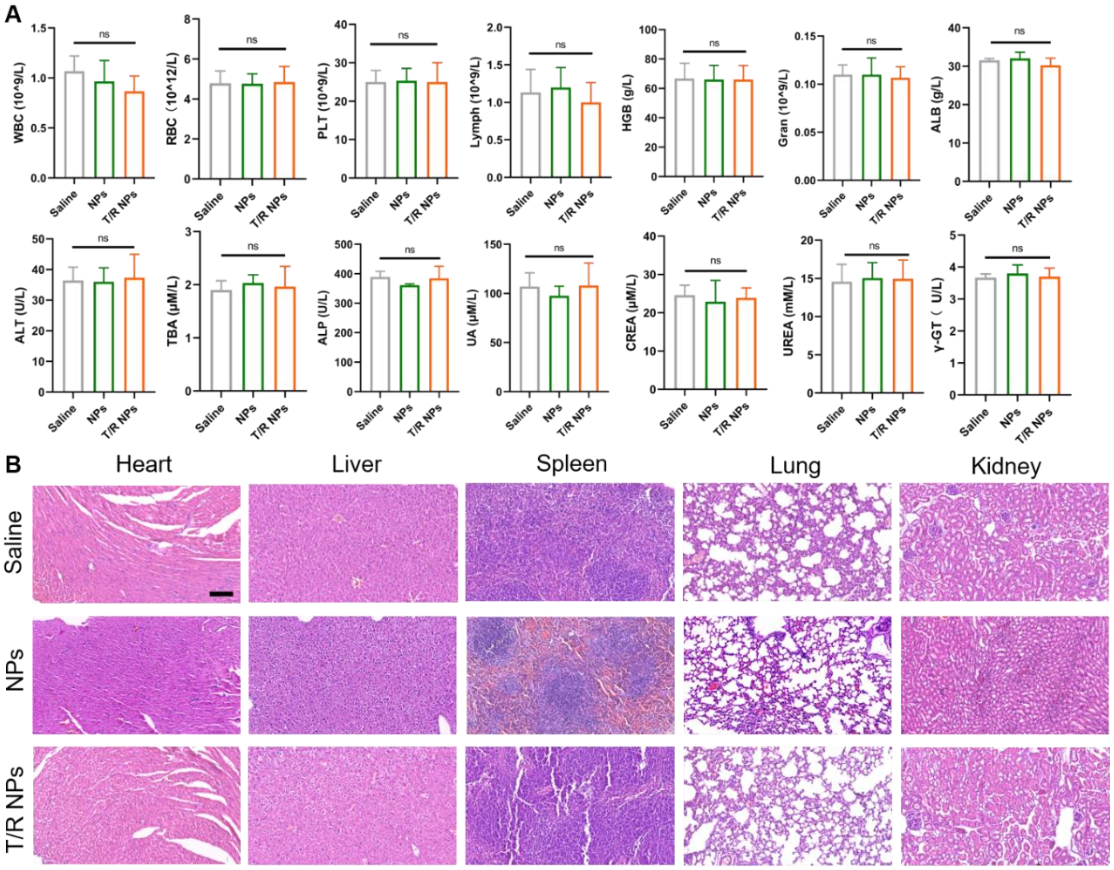

Figure S11. Toxicological evaluations and H&E staining after one-month treatment. (A) Routine blood and biochemical assays. RBCs, WBCs, PLT, GRAN, HGB, ALT, ALP, ALB,γ-GT, CREA, UREA, UA, and TBA. (B) H&E-stained images of main organs from mice (Scale bar: 100μm). Data in (A) are mean ± SD (*n* = 3).

Supplemental Table

Table S1. GPC characterization of copolymers.

**Polymer type**	**Number Average Molecular Weight (Mn)**	**Weight average molecular weight (Mw)**	**Mw/Mn**
$PLGA_{5000}$-Pep-PEG_{2000}	8.01 kDa	13.65 kDa	1.70
$PLGA_{5000}$- PEG_{2000}-c(RGDfC)	7.16 kDa	14.12 kDa	1.97

Table S2. Drug loading and encapsulation efficiency of RAP@T/R NPs.

**p1 : p2 :RAP (%w/w)**	**DLC ± SD (%)**	**DEE ± SD (%)**
5:5:10	9.26 ± 1.68	9.72 ± 1.76
10:10:10	15.03 ± 3.04	30.07 ± 6.09
20:20:10	14.62 ± 1.23	58.49 ± 4.94

Abbreviations: p1, PLGA-Pep-PEG; p2, PLGA-PEG-c(RGDfC); DLC, drug loading content; DEE, drug encapsulation efficiency; SD, standard deviation.
